# Effectiveness and safety of skull base-peripheral acupuncture for post-stroke cognitive impairment: a systematic review and meta-analysis of randomized controlled trials

**DOI:** 10.3389/fneur.2026.1790835

**Published:** 2026-04-30

**Authors:** Yinyi Xiong, Yi Chen, Xiaorong Zhang

**Affiliations:** Jiujiang University Affiliated Hospital, Jiujiang, China

**Keywords:** meta-analysis, post-stroke cognitive impairment, randomized controlled trials, skull base-peripheral acupuncture, systematic review

## Abstract

**Objective:**

To evaluate the efficacy and safety of skull base-peripheral (SBP) acupuncture in patients with post-stroke cognitive impairment (PSCI).

**Methods:**

We searched eight Chinese and English databases from inception to July 2025 for randomized controlled trials (RCTs) comparing SBP acupuncture with a control (no or sham acupuncture) in PSCI patients. Two reviewers independently screened studies, extracted data, and assessed outcomes including clinical effective rate, Mini-Mental State Examination (MMSE), Montreal Cognitive Assessment (MoCA), and activities of daily living (ADL). Data were analyzed using RevMan 5.3 and STATA 18.

**Results:**

Twenty-four RCTs involving 1,962 patients were included. Meta-analysis showed that SBP acupuncture was significantly superior to control in improving clinical effective rate (OR = 3.84, 95% CI: 2.79–5.27, *p* < 0.00001), MMSE scores (MD = 2.86, 95% CI: 2.02–3.71, *p* < 0.00001), MoCA scores (MD = 2.21, 95% CI: 1.63–2.80, *p* < 0.00001), and ADL (SMD = 1.62, 95% CI: 1.02–2.22, *p* < 0.00001), despite substantial heterogeneity. Subgroup analyses suggested enhanced benefits when SBP was part of combination therapy or for treatment durations under three months. No serious adverse events were reported.

**Conclusion:**

SBP acupuncture may have potential benefits for cognitive function in patients with PSCI, particularly when combined with other therapies or used for shorter durations. However, these cognitive findings should be considered preliminary due to methodological limitations and high heterogeneity. The benefits for ADL outcomes remain uncertain due to very low certainty of evidence and potential publication bias. The safety profile appeared acceptable based on available data. Further large-scale, multicenter, registered RCTs with rigorous methodologies and long-term follow-up are warranted.

**Systematic review registration:**

https://www.crd.york.ac.uk/PROSPERO/view/CRD420251142773, identifier CRD420251142773.

## 1 Introduction

Post-stroke cognitive impairment (PSCI) is a prevalent and debilitating complication following ischemic or hemorrhagic stroke, characterized by decline across multiple cognitive domains, including orientation, attention, memory, verbal fluency, and executive function. In addition to cognitive deficits, PSCI is often accompanied by neuropsychiatric symptoms and a reduced quality of life. These manifestations not only impede neurological recovery but also serve as a significant predictor of poor long-term outcomes, placing a substantial burden on families and society. At present, clinical management of PSCI remains challenging due to a lack of targeted and effective protocols (1). Given the growing global burden of stroke, developing effective and safe therapies for PSCI represents an urgent public health priority.

Pharmacological strategies developed for Alzheimer’s disease (AD) are often repurposed for PSCI, yet they typically yield little benefit while posing an elevated risk of adverse effects over time. In contrast, non-pharmacological interventions, especially acupuncture, have been increasingly recognized for their potential benefits (e.g., effectiveness, safety, accessibility and low cost) worldwide (2). Although the efficacy of acupuncture for PSCI has been supported by numerous meta-analyses comparing experimental groups receiving acupuncture-based therapies with control groups receiving sham acupuncture, blank control, or other non-acupuncture therapies (3–14), further validation regarding optimal acupoint combinations remains warranted, as its clinical application is largely constrained by their diversity.

In traditional Chinese medicine (TCM), acupoint selection is grounded in its theoretical framework. Within this system, PSCI is classified under traditional disorder domains such as “forgetfulness,” “mental dullness,” and “dementia.” Its pathogenesis is characterized by an excess of pathogenic factors coexisting with an underlying deficiency of vital energy (15, 16). Notably, the etiology of PSCI differs from that of dementia caused primarily by aging. Instead, PSCI develops following a stroke, which induces a pathology marked by dysfunction of the zang-fu organs and disruption in the flow of qi and blood. According to TCM literature, static blood and clouded phlegm are considered the predominant pathogenic factors in the early stages of PSCI, as they obstruct the circulation of qi and blood, thereby impairing the nourishment of the brain. In the later stages, the pathological picture shifts, with the depletion of kidney essence and the resulting marrow deficiency becoming progressively more evident.

Since the skull base-peripheral (SBP) acupoints are located at pivotal junctions where the qi of multiple meridians converges and infuses into the brain, they appear particularly advantageous for activating the mind and nourishing the sea of marrow. Previous meta-analyses have reported favorable findings of scalp acupuncture and the governor vessel acupoints for PSCI (17, 18). Although the SBP acupoints partially overlap with both scalp acupuncture and governor vessel acupoints, their unique efficacy for PSCI warrants systematic evaluation. A great many trials have investigated them; however, the consistency and generalizability of their findings are compromised by substantial heterogeneity in study design, sample size, and treatment protocols (19–25).

Accordingly, this systematic review and meta-analysis aims to evaluate the effectiveness and safety of SBP acupuncture for PSCI by comparing SBP acupuncture with blank control or sham acupuncture, thereby providing evidence-based information to support the optimization of acupoint protocols. Furthermore, our study represents an improvement over the poor methodological quality that has characterized previous systematic reviews on acupuncture for PSCI (26).

## 2 Methods

This review was conducted and reported following the Preferred Reporting Items for Systematic Reviews and Meta-analyses (PRISMA) guidelines. Prior to study initiation, a detailed protocol was prospectively registered on the PROSPERO international prospective register of systematic reviews with the registration number is CRD420251142773. No significant deviations were made from the approved protocol during the review process.

### 2.1 Search strategy

This systematic review searched the following eight databases from their inception to July 31, 2025: Chinese National Knowledge Infrastructure (CNKI), Wanfang, VIP Chinese Journal Service Platform, China Biology Medicine disc (CBM), PubMed, Web of Science, Cochrane Library, and Embase. To minimize the risk of omitting eligible studies, we also manually screened the reference lists of relevant systematic reviews. A comprehensive search strategy was developed using a combination of Medical Subject Headings (MeSH) and free-text terms, such as cognitive dysfunction, cognitive disorder, cognitive impairment, stroke, cerebrovascular accident, acupuncture, acupuncture therapy, acupuncture points. The PubMed (English) and CBM (Chinese) strategies served as templates, which were subsequently translated and modified for application in all other databases. The full search strategies are provided in Supplementary Tables 1–8.

### 2.2 Inclusion criteria

The study eligibility was defined according to the PICOS (Population, Intervention, Comparator, Outcomes, Study design) framework: (1) Population: Patients diagnosed with PSCI, with no restrictions regarding age, gender, education, disease duration, or case source. (2) Intervention: The experimental intervention consisted of standard treatment plus acupuncture therapy (e.g., manual acupuncture, electroacupuncture, or warm needle moxibustion) involving at least two SBP acupoints. (3) Comparator: Control groups received either conventional treatment (e.g., routine care, cognition-enhancing medications, cognitive training, or physical therapy) or sham acupuncture. (4) Outcomes: Primary outcomes were the clinical effective rate, Mini-Mental State Examination (MMSE), and Montreal Cognitive Assessment (MoCA). Secondary outcomes included Activities of Daily Living (ADL), while adverse events were recorded for safety evaluation. (5) Study design: Only RCTs were included.

### 2.3 Exclusion criteria

Studies were excluded if they met any of the following criteria: (1) Studies that were not clinical RCTs for human, including animal experiments and others. (2) Studies with suspected scientific misconduct or serious concerns regarding data integrity. (3) Duplicate publications or studies that presented overlapping datasets. (4) Studies for which the complete text could not be retrieved or which contained incomplete outcome data.

### 2.4 Study collection process

Duplicate records were removed using the automatic deduplication feature in EndNote 21, supplemented by a manual review to ensure completeness. Two researchers (YX and YC) then independently screened the remaining records and extracted data from the finally included studies. The screening process began with a review of titles and abstracts to exclude studies that clearly did not meet the eligibility criteria. The full texts of the remaining articles were then assessed to determine final inclusion. Only studies fulfilling all predefined eligibility criteria were included in the analysis. The standard data extraction form was predefined, including the first author, publication year, study methodology, patient characteristics, intervention parameters, outcome measures, and adverse events. All extracted data were documented in a standardized Microsoft Excel (2019) spreadsheet. Any disagreement between the two reviewers was referred to a third researcher (XZ) for a final decision.

### 2.5 Risk of bias assessment

The methodological quality of the included studies was independently assessed by two researchers (YX and YC) using the Cochrane Risk of Bias (ROB) tool. The evaluation covered seven domains: random sequence generation, allocation concealment, blinding of participants and personnel, blinding of outcome assessment, incomplete outcome data, selective reporting, and other potential sources of bias. Each domain was judged as “low risk,” “high risk,” or “unclear risk” in accordance with the tool’s handbook. Any disagreements in assessment were resolved through discussion with a third researcher (XZ).

### 2.6 Statistical analysis

This study conducted all meta-analyses using the RevMan 5.3 and STATA 18 software. Dichotomous outcomes were analyzed using odds ratios (ORs), while continuous outcomes were analyzed using mean differences (MDs) or standardized mean differences (SMDs). All effect estimates are reported with 95% confidence intervals (CIs). The pooled effects were calculated using either fixed-effects or random-effects model. Model selection was guided by the degree of statistical heterogeneity, as measured by the *Q* statistic (with a significance threshold of *p* < 0.05) and *I*^2^ statistic. A fixed-effects model was employed when no significant heterogeneity was detected (*p* ≥ 0.05 and *I*^2^ ≤ 50%); otherwise, a random-effects model was used. Potential sources of heterogeneity were explored through subgroup analyses. The robustness of findings was assessed using sensitive analyses, and potential publication bias was evaluated through visual inspection of funnel plots and Egger’s test. PET-PEESE analyses were performed using the metafor package in R (version 4.5.2).

## 3 Results

### 3.1 Study characteristics

**Figure 1:**
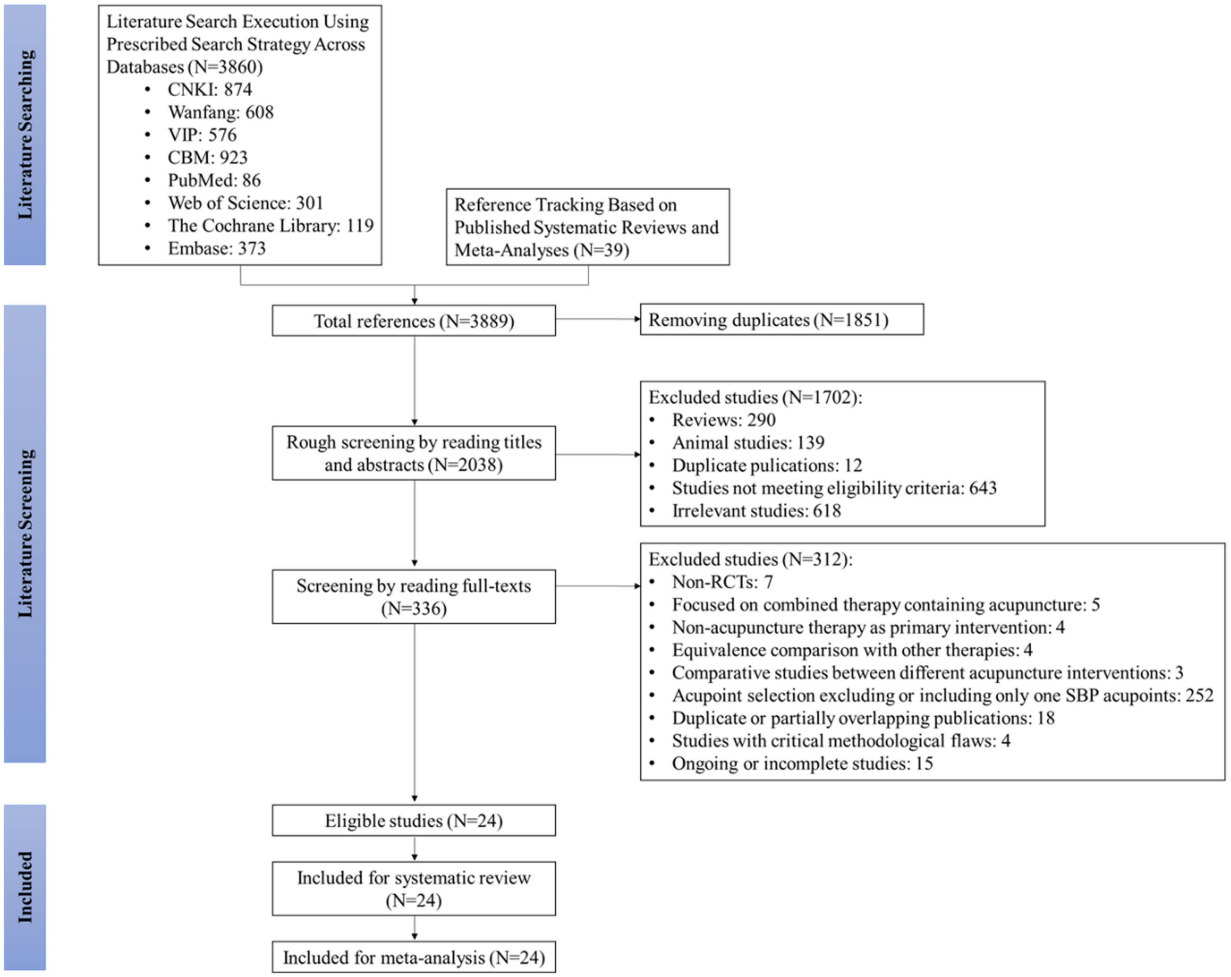
Literature screening flowchart for SBP acupuncture in patients with PSCI.

Our systematic search initially identified 3,899 records. After removing 1,851 duplicates, 2,038 records were screened based on titles and abstracts, yielding 336 articles for full-text review. Following a detailed assessment, 24 studies (19–25, 27–43) met all eligibility criteria and were included in the meta-analysis (Figure 1). The final pool consisted of 22 Chinese-language (19–25, 27–41) and two English-language (42, 43) publications.

All included RCTs were conducted in China. Enrolling a total of 1,962 patients (978 in intervention groups), the studies predominantly featured a single-center, parallel-group design. Except for one multicenter and three-arm trial (27), and two that used sham acupuncture controls (19, 41), the remaining studies employed a blank control design. The fundamental characteristics of the included studies are summarized in Tables 1, 2.

**Table 1:** Methodological and baseline characteristics of included studies.

Eligible studies	Sample size (T/C, N)			Methodology of RCTs			Stroke type	Mean age (years)	Gender (Male/Female, *N*)	Education level	Disease duration
	Recruitment	Drops	Analysis	Random sequence generation	Allocation concealment	Blinding					
Yu et al. (28)	54	3	25/26	Not reported	Not reported	Not reported	Ischemic stroke	T: 63.3 C: 57.7	T: 14/11 C: 15/11	Not reported	T: 79.9 days C: 76.5 days
Li (25)	60	0	30/30	Random number table	Not reported	Not reported	Ischemic stroke	50–55 years old/56–70 years old /71–75 years old T: 7/14/9 C: 6/16/8	T: 14/16 C: 15/15	Not reported	<1 month/1–3 months/3–6 months T: 4/15/11 C: 3/15/12
Wang et al. (42)	126	2/3	40/39	Not reported	Sealed envelopes	Outcome assessors and data analysts	Ischemic stroke	T: 65.2 ± 7.1 C: 60.6 ± 6.7 Incomparable	T: 26/14 C: 26/13	Not reported	Not reported
Liu et al. (43)	168	0	84/84	Not reported	Not reported	Not reported	Not reported	T: 55 ± 7 C: 56 ± 9	T: 54/30 C: 52/32	T: 6.33 ± 2.48 years C: 6.47 ± 1.96 Year	T: 2.9 ± 0.7 years C: 3.2 ± 0.9 Years
Zhang et al. (29)	84	0	42/42	Lottery drawing method	Not reported	Not reported	Not reported	T: 63.28 ± 10.68 C: 63.07 ± 10.59	T: 27/15 C: 28/14	Not reported	T: 2.14 ± 1.22 months C: 2.31 ± 1.19 months
Xiang et al. (31)	80	0	40/40	Not reported	Not reported	Not reported	Not reported	T: 62.2 ± 4.6 C: 61.9 ± 4.6	T: 24/16 C: 23/17	Not reported	T: 2.2 ± 0.5 months C: 2.2 ± 0.5 months
Wang et al. (30)	118	0	59/59	Random number table	Not reported	Not reported	Ischemic stroke	T: 68.88 ± 3.64 C: 67.71 ± 3.02	T: 36/23 C: 32/27	Not reported	T: 2.09 ± 0.63 months C: 2.3 ± 0.65 months
Sha et al. (33)	60	0	20/20	Random number table	Not reported	Not reported	Ischemic stroke	Comparable but no specific data provided	Comparable but no specific data provided	Comparable but no specific data provided	Comparable but no specific data provided
Wen et al. (34)	60	0	30/30	Random number table	Not reported	Not reported	Not reported	T: 64 ± 7.18 C: 63.7 ± 6.98	T: 14/16 C: 17/13	Not reported	T: 5.7 ± 1.92 months C: 5.3 ± 1.9 months
Chen (32)	80	0	40/40	Random number table	Not reported	Not reported	Ischemic stroke	T: 56.85 ± 17.12 C: 56.95 ± 18.09	T: 22/18 C: 21/19	Not reported	T: 3.72 ± 2.57 months C: 3.54 ± 2.74 months
Cao (22)	92	0	30/31	Random number table	Not reported	Not reported	Not reported	T: 54.47 ± 12.21 C: 56.26 ± 12.16	T: 14/16 C: 17/14	Not reported	T: 3.47 ± 1.28 months C: 3.58 ± 1.12 months
Xu (37)	90	0	45/45	Random number table	Not reported	Not reported	Ischemic stroke, hemorrhagic stroke	T: 67.85 ± 1.91 C: 68.02 ± 2.03	T: 28/17 C: 30/15	Not reported	T: 1.94 ± 0.34 months C: 2.05 ± 0.37 months
Shang et al. (36)	60	0	30/30	Random number table	Not reported	Not reported	Ischemic stroke	T: 57 ± 6.8 C: 56 ± 6.6	T: 19/11 C: 18/12	Primary school/Secondary school/University T: 2/18/10 C: 3/16/11	T: 2.9 ± 1.3 months C: 2.8 ± 1.5 months
Xu (37)	60	0	30/30	Computer-generated sequences	Not reported	Outcome assessors and data analysts	Ischemic stroke	T: 61.9 ± 8.61 C: 60.87 ± 8.98	T: 17/13 C: 19/11	T: 6.57 ± 2.64 years C: 6.97 ± 2.48 years	T: 70.8 ± 8.14 days C: 68.37 ± 13.61 days
Bi (35)	102	0	51/51	Not reported	Not reported	Not reported	Ischemic stroke	T: 64.2 ± 8.7 C: 63.9 ± 8.9	T: 30/21 C: 30/21	Not reported	T: 1.4 ± 0.5 months C: 1.2 ± 0.4 months
Li and Pan (39)	80	0	40/40	Random number table	Not reported	Not reported	ischemic stroke, hemorrhagic stroke	T: 68.6 ± 2.5 C: 67.9 ± 3.7	T: 29/11 C: 32/8	T: 2.1 ± 6.5 years C: 2.5 ± 6.3 years	T: 2.94 ± 0.95 months C: 3.04 ± 0.75 months
Yang (27)	180	5/2	55/58	Central randomization system	Central randomization system	Participants, outcome assessors and data analysts	Ischemic stroke	T: 62 (55, 68) C: 60 (52.75, 67.25)	T: 39/16 C: 40/18	Illiterate/Primary school/Junior secondary school/Senior secondary school/University T: 3/6/25/15/6 C: 3/8/24/16/7	<3 months/3–6 months T: 46/9 C: 51/7
Li et al. (19)	76	4/4	34/34	Computer-generated sequences	Sealed envelopes	Outcome assessors and data analysts	Ischemic stroke, hemorrhagic stroke	T: 69 ± 8 C: 67 ± 9	T: 21/13 C: 22/12	Illiterate/Primary school/Secondary school and above T: 1/6/27 C: 1/4/29	Not reported
Fan et al. (38)	90	4/2	41/43	Random number table	Not reported	Not reported	Ischemic stroke	T: 61.41 ± 10.88 C: 62.89 ± 8.74	T: 22/19 C: 25/18	Not reported	T: 8.67 ± 2.08 weeks C: 8.52 ± 2.14 weeks
Xing (20)	82	2/2	39/39	Random number table	Not reported	Rehabilitation therapists, outcome assessors and data analysts	Ischemic stroke, hemorrhagic stroke	T: 65.95 ± 5.69 C: 63.72 ± 6.97	T: 21/18 C: 19/20	Illiterate/Primary school/Secondary school and above T: 2/10/27 C: 3/8/28	T: 67.18 ± 8.84 days C: 65.87 ± 10.54 days
Feng (23)	124	0	62/62	Random number table	Not reported	Not reported	Ischemic stroke	T: 53.09 ± 4.48 C: 53.12 ± 4.51	T: 35/27 C: 37/25	Not reported	T: 1.85 ± 0.41 months C: 1.79 ± 0.39 months
Xie et al. (40)	80	0	40/40	Random number table	Not reported	Not reported	Ischemic stroke, hemorrhagic stroke	T: 63.24 ± 11.21 C: 62.55 ± 10.41	T: 21/19 C: 19/21	Not reported	T: 3.24 ± 0.51 years C: 3.09 ± 0.48 years
Han et al. (24)	70	0/1	35/35	Random number table	Not reported	Not reported	Ischemic stroke	T: 60.14 ± 7.32 C: 60.85 ± 7.34	T: 19/16 C: 18/17	Primary school/Secondary school and above T: 7/28 C: 8/27	T: 12.17 ± 4.59 weeks C: 11.25 ± 3.87 weeks
Chi et al. (41)	72	1/1	36/36	Random number table	Not reported	Participants	Ischemic stroke	T: 62.86 ± 8.35 C: 61.37 ± 8.03	T: 24/12 C: 25/11	Not reported	T: 57.91 ± 2.37 days C: 58.54 ± 2.88 days

**Table 2:** Basic characteristics of eligible studies: interventions, outcomes, adverse events.

Eligible studies	Grouping		Acupuncture technique	Acupoints selection	Treatment duration	Outcomes	Adverse events
	C	T					
Yu et al. (28)	Routine care + nimodipine	C + acupuncture (SBP plus governor vessel-regulating acupoints)	Manual acupuncture	Baihui (GV20), Shenting (GV24), Fengfu (GV16), Yintang (EX-HN3), Dazhui (GV14), Quchai (BL4), Fengchi (GB20), Neiguan (PC6), Hegu (LI4), Zusanli (ST36), Sanyinjiao (SP6)	6 months	Clinical effective rate, ADL (MBI)	Not reported
Li (25)	Routine care + donepezil	C + acupuncture (SBP acupoints)	Electroacupuncture	Fengchi (GB20), Gongxue (Extra), Yiming (Extra)	3 months	Clinical effective rate, MMSE, MoCA	Two cases of dizziness and nausea occurred in the treatment group
Wang et al. (42)	Routine care + nimodipine	C + acupuncture (SBP plus intelligence-enhancing and mind-regulating acupoints)	Manual acupuncture	Baihui (GV20), Sishengcong (EX-HN1), Sibai (ST2), Fengchi (GB20), Wangu (GB12), Tianzhu (BL10), Renzhong (GV26), Shenmen (HT7), Neiguan (PC6), Fenglong (ST40), Sanyinjiao (SP6), Taichong (LR3)	3 months	Clinical effective rate, MoCA	No significant adverse reactions were observed
Liu et al. (43)	Routine care + donepezil	C + acupuncture (SBP plus intelligence-enhancing and mind-regulating acupoints)	Manual acupuncture	Shenting (GV24), Fengchi (GB20), Wangu (GB12), Tianzhu (BL10), Sishengcong (EX-HN1), Yintang (EX-HN3), Renzhong (GV26), Neiguan (PC6), Shenmen (HT7), Baihui (GV20)	8 weeks	MMSE	Not reported
Zhang et al. (29)	Routine care	C + acupuncture (SBP plus governor vessel-regulating acupoints)	Manual acupuncture	Baihui (GV20), Fengfu (GV16), Yamen (GV15), Shenting (GV24), Renzhong (GV26), Dazhui (GV14), Cervical Jiaji (EX-B2)	4 weeks	Clinical effective rate, MMSE	Not reported
Xiang et al. (31)	Cognitive training + nimodipine	C + acupuncture (SBP plus scalp acupuncture therapy)	Manual acupuncture	Precentral Region, Frontal Region, Sishengcong (EX-HN1), Baihui (GV20), Fengchi (GB20), Fengfu (GV16), Yiming (Extra), Gongxue (Extra), Taichong (LR3), Guanyuan (CV4), Shenshu (BL23)	56 times	Clinical effective rate, MMSE, ADL (BI)	Not reported
Wang et al. (30)	Routine care	C + acupuncture (SBP plus governor vessel-regulating acupoints)	Manual acupuncture	Baihui (GV20), Shenting (GV24), Fengfu (GV16), Fengchi (GB20), Dazhui (GV14), Cervical Jiaji (EX-B2), Quchi (LI11), Neiguan (PC6), Fengshi (GB31), Zusanli (ST36), Yanglingquan (GB34), Sanyinjiao (SP6), Xuehai (SP10)	30 days	Clinical effective rate, MMSE	Nine cases in the control group and eight cases in the treatment group experienced adverse events, primarily manifested as nausea, vomiting, dyspepsia, abdominal pain, diarrhea, and dizziness
Sha et al. (33)	Routine care + nimodipine	C + acupuncture (SBP plus intelligence-enhancing and mind-regulating acupoints)	Manual acupuncture	Renzhong (GV26), Baihui (GV20), Sishengcong (EX-HN1), Fengchi (GB20), Wangu (GB12), Tianzhu (BL10), Sibai (ST2), Neiguan (PC6), Taichong (LR3), Fenglong (ST40), Shenmen (HT7), Sanyinjiao (SP6)	12 weeks	MoCA	No significant adverse reactions were observed
Wen et al. (34)	Routine care + cognitive training	C + acupuncture (SBP plus governor vessel-regulating acupoints)	Manual acupuncture	Renzhong (GV26), Yintang (EX-HN3), Shenting (GV24), Baihui (GV20), Fengfu (GV16), Yamen (GV15), Yaoyangguan (GV3), Mingmeng (GV4)	40 times	Clinical effective rate, MMSE, ADL (MBI)	Not reported
Chen (32)	Routine care + cognitive training + physical therapy	C + acupuncture (SBP plus scalp acupuncture therapy)	Manual acupuncture	Contralateral anterior temporal–parietal line, posterior temporal–parietal line, and mid-frontal line; ipsilateral anterior temporal line and posterior temporal line; Baihui (GV20), Sishengcong (EX-HN1), Fengfu (GV16), Fengchi (GB20), Yamen (GV15), Tongtian (BL7)	1 month	MoCA, MMSE, ADL (MBI)	Not reported
Cao (22)	Routine care + cognitive training + physical therapy	C + acupuncture (SBP acupoints)	Manual acupuncture	Fengchi (GB20), Gongxue (Extra), Yiming (Extra)	10 times	Clinical effective rate, MoCA	Not reported
Xu et al. (21)	Routine care + cognitive training	C + acupuncture (SBP acupoints)	Manual acupuncture	Fengchi (GB20), Fengfu (GV16), Xiangsihua (Extra), Dazhui (GV14)	8 weeks	MMSE, ADL (BI)	Not reported
Shang et al. (36)	Routine care + physical therapy	C + acupuncture (SBP plus scalp acupuncture therapy)	Manual acupuncture	Fengchi (GB20), Fengfu (GV16), Baihui (GV20), Emotional Area, Dizziness and Hearing Area, Yiming (Extra), Gongxue (Extra)	4 weeks	MoCA	Not reported
Xu (37)	Routine care + donepezil	C + acupuncture (SBP plus governor vessel-regulating acupoints)	Manual acupuncture	Baihui (GV20), Shenting (GV24), Fengfu (GV16), Yamen (GV15), Dazhui (GV14), Yaoyangguan (GV3), Mingmeng (GV4), Zhiyang (GV9), Shendao (GV11), Cervical Jiaji (EX-B2)	8 weeks	MoCA, MMSE	Mentioned but no specific data provided
Bi (35)	Routine care + oxiracetam + cognitive training	C + acupuncture (SBP plus intelligence-enhancing and mind-regulating acupoints)	Manual acupuncture	Baihui (GV20), Sishengcong (EX-HN1), Sibai (ST2), Fengchi (GB20), Renzhong (GV26), Wangu (GB12), Tianzhu (BL10), Shenmen (HT7), Neiguan (PC6), Sanyinjiao (SP6), Taichong (LR3), Fenglong (ST40)	6 weeks	Clinical effective rate, MoCA, MMSE, ADL (ADL)	Not reported
Li and Pan (39)	Routine care + cognitive training	C + acupuncture (SBP plus scalp acupuncture therapy)	Manual acupuncture	Shenting (GV24), Touwei (ST8), Toulinqi (GB15), Baihui (GV20), Baihuipang, Shuaigu (GB8), Jiaosun (SJ20), Tianchong (GB9), Luxi (SJ19), Qubin (GB7), Ermen (SJ21), Naohu (GV17), Fengfu (GV16), Yuzhen (BL9), Tianzhu (BL10)	4 weeks	MoCA, ADL (MBI)	Not reported
Yang (27)	Routine care + sham acupuncture (proximal points)	Routine care+ acupuncture (SBP plus intelligence-enhancing and mind-regulating acupoints)	Manual acupuncture	Baihui (GV20), Sishengcong (EX-HN1), Renzhong (GV26), Sibai (ST2), Fengchi (GB20), Wangu (GB12), Tianzhu (BL10), Neiguan (PC6), Shenmen (HT7), Fenglong (ST40), Sanyinjiao (SP6), Taichong (LR3)	12 weeks	MoCA, MMSE	No significant adverse reactions were observed
Li et al. (19)	Sham acupuncture (distal points)	Acupuncture (SBP acupoints)	Manual acupuncture	Fengchi (GB20), Wangu (GB12), Tianzhu (BL10), Yamen (GV15), Baihui (GV20)	4 weeks	Clinical effective rate, MoCA, MMSE, ADL (BI)	One case of subcutaneous hemorrhage occurred in the control group; one case of needle fainting occurred in the treatment group
Fan et al. (38)	Oxiracetam	C + acupuncture (SBP plus scalp acupuncture therapy)	Manual acupuncture	Fengchi (GB20), Fengfu (GV16), Baihui (GV20), Emotional Area, Dizziness and Hearing Area, Yiming (Extra), Gongxue (Extra)	4 weeks	Clinical effective rate, MoCA, MMSE, ADL (BI)	Not reported
Xing (20)	Routine care + cognitive training	C + acupuncture (SBP acupoints)	Electroacupuncture	Fengchi (GB20), Tianzhu (BL10), Gongxue (Extra), Yifeng (SJ17), Fengfu (GV16)	8 weeks	Clinical effective rate, MoCA, MMSE, ADL (MBI)	One case of needle sticking occurred in the treatment group
Feng (23)	Cognitive training	C + acupuncture (SBP acupoints)	Manual acupuncture	Fengchi (GB20), Fengfu (GV16), Xiangsihua (Extra)	8 weeks	MMSE, ADL (BI)	Not reported
Xie et al. (40)	Cognitive training	C + acupuncture (SBP plus governor vessel-regulating acupoints)	Manual acupuncture	Baihui (GV20), Fengfu (GV16), Dazhui (GV14), Yamen (GV15), Renying (ST9), Qichong (ST30), Zusanli (ST36), Dazhu (BL11), Shangjuxu (ST37), Xiajuxu (ST39)	2 weeks	MMSE, MoCA	Not reported
Han et al. (24)	Routine care + donepezil	C + acupuncture (SBP acupoints)	Electroacupuncture	Fengchi (GB20), Gongxue (Extra)	4 weeks	Clinical effective rate, MoCA, MMSE, ADL (ADL)	One case of hematoma occurred in the treatment group
Chi et al. (41)	Routine care + cognitive training + sham acupuncture (proximal points)	Routine care + cognitive training + acupuncture (SBP plus intelligence-enhancing and mind-regulating acupoints)	Manual acupuncture	Neiguan (PC6), Renzhong (GV26), Baihui (GV20), Sishengcong (EX-HN1), Fengchi (GB20), Wangu (GB12), Tianzhu (BL10), Sibai (ST2), Shenmen (HT7), Fenglong (ST40), Sanyinjiao (SP6), Taichong (LR3)	12 weeks	Clinical effective rate, MoCA, MMSE	No significant adverse reactions were observed

T, SEP acupuncture group; C, control group.

The diagnostic criteria for PSCI were reviewed across all included studies. Most studies based their diagnosis on established clinical guidelines for cerebrovascular disease and cognitive disorders. Apart from three studies (23, 34, 38) where insufficient information prevented confirmation of a causal link between stroke and cognitive decline, all others clearly confirmed that their cases met the core diagnostic criteria for PSCI. Regarding stroke types, 14 studies (23–25, 27, 28, 30, 32, 33, 35–38, 41, 42) exclusively enrolled ischemic stroke patients, five (19–21, 39, 40) included both ischemic and hemorrhagic cases, and the remaining five (22, 29, 31, 34, 43) did not report specific stroke subtypes.

All but one study (33) reported detailed baseline characteristics, including mean age, sex distribution, and disease duration. Pooled data indicated that approximately 60% of the participants were male, with ages predominantly ranging from 40 to 80 years. With the exception of two studies (40, 43) where the disease durations extended to 3 years, all other studies enrolled patients within 6 months post-stroke. Notably, one study (42) reported a significant age discrepancy, with the control group being younger than the intervention group. All remaining studies claimed baseline comparability between groups.

Acupuncture served as the core therapeutic modality across all included studies, delivered as electroacupuncture in three trials (20, 24, 25) and manual acupuncture in the remaining 21. Regarding therapeutic combinations, four studies (19, 27, 29, 30) utilized acupuncture as a standalone cognitive intervention, while the remaining 20 combined acupuncture with other therapies: eight studies (24, 25, 28, 33, 37, 38, 42, 43) supplemented treatment with cognitive-enhancing medications, seven (20, 21, 23, 34, 39–41) incorporated cognitive training, one (36) included physical therapy and four studies (22, 31, 32, 35) applied at least two additional therapeutic modalities. The selection and number of acupoints varied across studies. Seven studies (19–25) used SBP acupoints exclusively, while other studies combined SBP acupoints with other acupoint combinations. Among these, six studies (27, 33, 35, 41–43) supplemented with acupoints known for their mind-activating and intelligence-enhancing properties, such as Baihui (GV20) and Neiguan (PC6); six (28–30, 34, 37, 40) included acupoints for regulating the governor vessel, such as Baihui (GV20) and Dazhui (GV14); and five (31, 32, 36, 38, 39) incorporated scalp acupuncture. Regarding treatment duration, two studies (22, 40) had courses shorter than 2 weeks, 16 studies (19–21, 23, 24, 29–32, 34–39, 43) lasted 1 to 2 months, and six (25, 27, 28, 33, 41, 42) extended to 3 to 6 months.

All included studies reported positive effects of acupuncture intervention on cognitive function in patients with PSCI. Relevant outcome measures—including clinical effective rate, MMSE scores, MoCA scores, and activities of daily living—were assessed immediately following the completion of treatment. However, only two studies (27, 42) conducted follow-up assessments to evaluate the long-term sustainability of these effects.

### 3.2 Assessment of ROB

Five studies (28, 31, 35, 42, 43) did not describe the specific randomization methods, while 19 had a low ROB about random sequence generation: 15 studies (20–25, 30, 32–34, 36, 38–41) utilized a random number table, two (19, 37) employed computer-generated sequences, one (29) used a lottery drawing method, one (27) implemented a central randomization system. Regarding allocation concealment, only two studies (19, 42) used sealed envelopes, one study (27) with central randomization naturally met the requirements, while the remaining studies did not mention any relevant measures.

Due to the nature of acupuncture therapy, blinding of acupuncturists was not feasible in any of the included studies, but one study (20) adopted blinding of rehabilitation therapists. Three studies (20, 37, 42) explicitly reported no blinding of participants, while three studies (19, 27, 41) attempted participant blinding using sham acupuncture. However, in one (19) of these studies, the sham acupuncture applied at locations substantially different from the SBP region, a methodological flaw that likely compromised blinding effectiveness as participants could have discerned the distinct therapeutic approaches between groups. Consequently, this study was judged to be at high ROB concerning participant blinding. In addition, five studies (19, 20, 27, 37, 42) maintained blinding of outcome assessors and data analysts.

**Figure 2:**
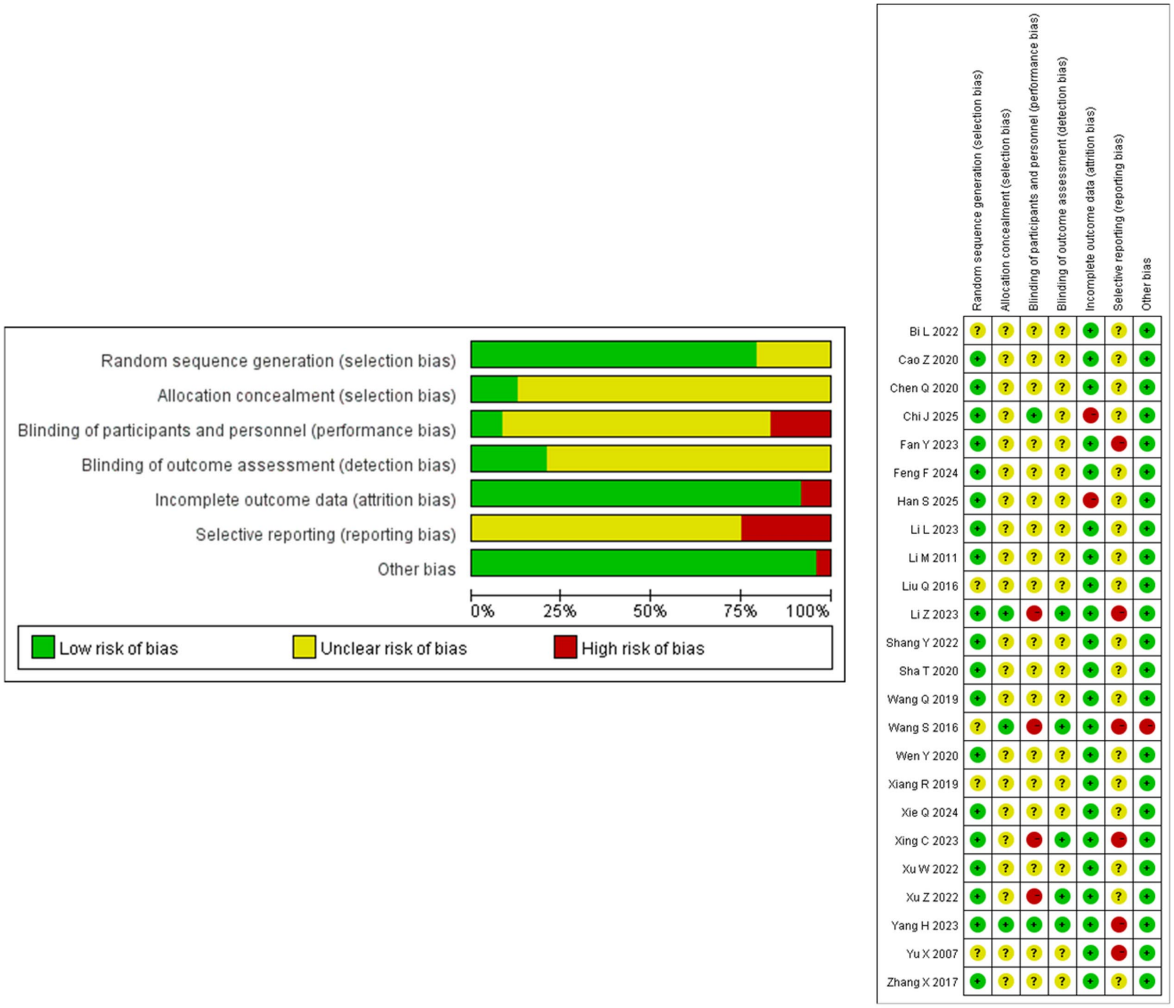
Risk of bias assessment for the included studies.

Participant dropouts were reported in eight studies (19, 20, 24, 27, 28, 38, 41, 42). Among these, four (19, 20, 27, 41) provided detailed information on dropout numbers and reasons by treatment group, one (28) reported only aggregate data without group-specific details, and the remaining three (24, 38, 42) did not specify the reasons for attrition. For handling missing data, two studies (24, 41) employed a complete-case (CC) analysis and were judged to be at high ROB due to incomplete outcome data. The other six studies (19, 20, 27, 28, 38, 42) utilized a per-protocol (PP) analysis, which also introduce a high ROB of reporting bias. In addition, one study (28) was judged to be at high risk of selective reporting bias as it only reported the clinical effective rate without providing pre-specified MMSE scores. The remaining studies were considered to have an unclear risk of reporting bias due to the absence of a prospective registration protocol. One study (42) exhibited significant baseline age imbalance (with the control group being younger than intervention group), resulting in a high ROB for underestimating the effect of SBP acupuncture. The results of the ROB assessment are shown in Figure 2.

### 3.3 Efficacy of SBP acupuncture for PSCI

#### 3.3.1 Clinical effective rate

**Figure 3:**
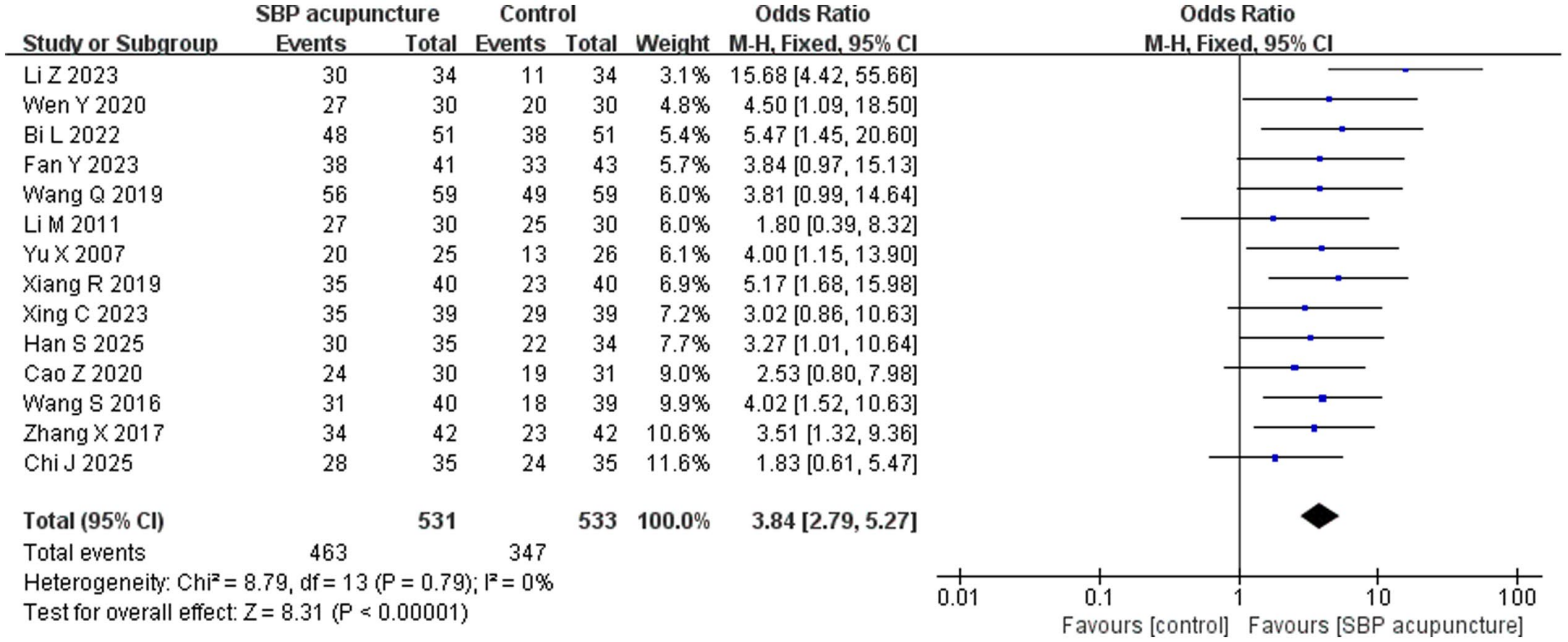
Forest plot of clinical effective rates.

Fourteen studies (19, 20, 22, 24, 25, 28–31, 34, 35, 38, 41, 42) involving 1,064 patients used the clinical effective rate as an outcome measure. Among these, 12 studies (19, 20, 22, 24, 25, 28, 31, 34, 35, 38, 41, 42) calculated the rate based on the improvement rate of MMSE or MoCA scores, while two studies (29, 30) directly used the net increase in MMSE scores for assessment. Heterogeneity testing indicated negligible heterogeneity among the studies (*χ*^2^ = 8.79, *p* = 0.79, *I*^2^ = 0%), justifying the use of a fixed-effects model for the meta-analysis. The pooled results demonstrated that SBP acupuncture was significantly superior to control interventions (OR = 3.84, 95% CI: 2.79–5.27, *Z* = 8.31, *p* < 0.00001; Figure 3).

#### 3.3.2 MMSE scores

**Figure 4:**
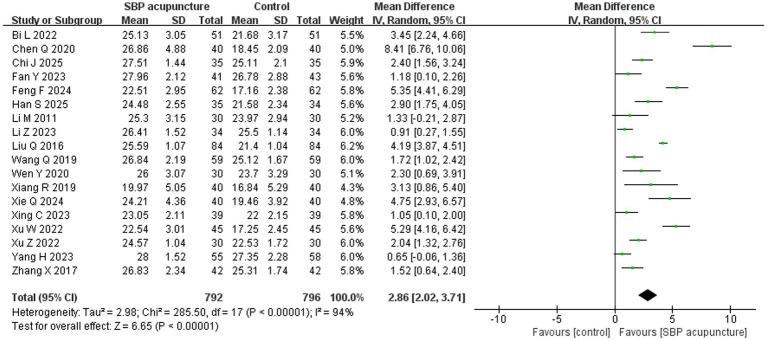
Forest plot of MMSE scores.

Eighteen studies (19–21, 23–25, 27, 29–32, 34, 35, 37, 38, 40, 41, 43) involving 1,588 patients used the MMSE score as an outcome measure. Data from the study by Yang et al. were converted to mean ± standard deviation using methods described by Luo et al. (44) and Wan et al. (45) before being included in the analysis. Significant heterogeneity was detected among the studies (*χ*^2^ = 285.5, *p* < 0.00001, *I*^2^ = 94%), and a random-effects model was therefore applied. The meta-analysis demonstrated that SBP acupuncture significantly improved MMSE scores compared with control interventions (MD = 2.86, 95% CI: 2.02–3.71, *Z* = 6.65, *p* < 0.00001; Figure 4).

To investigate potential sources of heterogeneity in MMSE outcomes, we performed subgroup analyses based on stroke type and four intervention dimensions: acupuncture technique, acupoint combination, treatment duration, and combination with other therapies (Table 3). The results demonstrated that substantial heterogeneity was significantly reduced when SBP acupuncture was administered as a standalone cognitive intervention (*χ*^2^ = 5.64, *p* = 0.13, *I*^2^ = 47%), indicating that the use of combination therapies represents a partial source of heterogeneity in MMSE outcomes.

**Table 3:** Subgroup analysis profiles of MMSE scores.

Items	No. of included studies	Heterogeneity (*I*^2^)	Overall effect (*p*)	Pooled effect sizes			Subgroup differences (*p* for interaction)
				MD	Lower 95% CI	Upper 95% CI	
Total							
MMSE	18	94	<0.00001	2.86	2.02	3.71	
Subgroup analysis by stroke type							
Ischemic stroke	10	93	<0.00001	2.88	1.74	4.01	1
Ischemic and hemorrhagic stroke	4	95	0.009	2.92	0.72	5.13	
Not reported	4	91	0.001	2.80	1.10	4.51	
Subgroup analysis by acupuncture technique							
Manual acupuncture	15	95	<0.00001	3.08	2.14	4.03	0.09
Electroacupuncture	3	68	0.004	1.76	0.55	2.97	
Subgroup analysis by acupoint combination							
Intervention using SBP acupoints exclusively	6	95	0.002	2.81	1.06	4.56	0.75
Combined with intelligence-enhancing and mind-regulating acupoints	4	97	0.004	2.67	0.84	4.51	
Combined with governor vessel-regulating acupoints	5	63	<0.00001	2.17	1.43	2.91	
Combined with scalp acupuncture therapy	3	96	0.08	4.23	−0.49	8.95	
Subgroup analysis by treatment duration							
Treatment courses shorter than 2 weeks	1	—	<0.00001	4.75	2.93	6.57	0.01
Treatment courses within 1–2 months	14	94	<0.00001	3.05	2.09	4.01	
Treatment courses within 3–6 months	3	79	0.02	1.46	0.23	2.69	
Subgroup analysis by combination with other therapies							
As a standalone cognitive intervention	4	47	<0.00001	1.18	0.68	1.67	0.004
Combined with cognitive-enhancing medications	5	93	0.0008	2.39	0.99	3.79	
Combined with cognitive training	6	92	<0.00001	3.5	1.92	5.09	
Combined with at least two additional therapeutic modalities	3	92	0.004	5.02	1.61	8.43	

Pooled effect estimates from subgroup analyses demonstrated that the therapeutic effect of SBP acupuncture was significantly moderated by treatment duration (*χ*^2^ = 9.26, *p* for interaction = 0.01), and combination with other therapies (*χ*^2^ = 13.37, *p* for interaction = 0.004). In contrast, stroke type (*χ*^2^ = 0.01, *p* for interaction = 1), acupuncture technique (*χ*^2^ = 2.84, *p* for interaction = 0.09), and acupoint combination (*χ*^2^ = 1.22, *p* for interaction = 0.75) did not show statistically significant interaction effects. Specifically, the subgroup analysis of SBP acupuncture served as a standalone cognitive intervention (MD = 1.18, 95% CI: 0.68–1.67) showed smaller improvements in MMSE scores compared to combinations with cognitive-enhancing medications (MD = 2.39, 95% CI: 0.99–3.79), cognitive training (MD = 3.5, 95% CI:1.92–5.09), or at least two additional therapeutic modalities (MD = 5.02, 95% CI: 1.61–8.43), suggesting superior efficacy when SBP acupuncture is integrated into a combination therapy regimen. Furthermore, shorter treatment durations demonstrated superior outcomes: courses shorter than 2 weeks (MD = 4.75, 95% CI: 2.93–6.57) and 1–2 months (MD = 3.05, 95% CI: 2.08–4.01) both showed greater improvement than the 3–6-month duration (MD = 1.46, 95% CI: 0.23–2.69), suggesting that extended treatment beyond 3 months may not provide additional therapeutic benefits for SBP acupuncture therapy.

#### 3.3.3 MoCA scores

**Figure 5:**
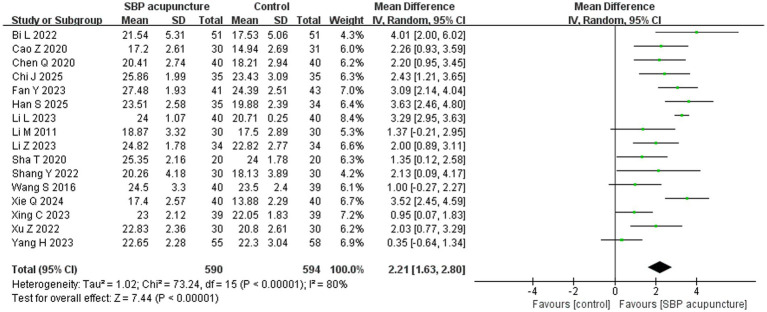
Forest plot of MoCA scores.

Sixteen studies (19, 20, 22, 24, 25, 27, 32, 33, 35–42) involving 1,184 patients used the MoCA score as an outcome measure. Data transformation methods were consistent with those described for the MMSE analysis. Significant heterogeneity was observed across studies (*χ*^2^ = 73.24, *p* < 0.00001, *I*^2^ = 80%), warranting the use of a random-effects model. The meta-analysis demonstrated that SBP acupuncture significantly improved MoCA scores compared to control conditions (MD = 2.21, 95% CI: 1.63–2.8, *Z* = 7.44, *p* < 0.00001; Figure 5).

The aforementioned subgroup analysis strategy was also applied to MoCA outcomes (Table 4). The results indicated that stratification by acupoint combination (using SBP acupoints exclusively: *χ*^2^ = 13.58, *p* = 0.009, *I*^2^ = 71%; combined with intelligence-enhancing and mind-regulating acupoints: *χ*^2^ = 13.94, *p* = 0.008, *I*^2^ = 71%; combined with governor vessel-regulating acupoints: *χ*^2^ = 3.13, *p* = 0.08, *I*^2^ = 68%; combined with scalp acupuncture therapy: *χ*^2^ = 3.83, *p* = 0.28, *I*^2^ = 22%) and by treatment duration (shorter than 2 weeks: *χ*^2^ = 2.1, *p* = 0.15, *I*^2^ = 52%; 1–2 months: *χ*^2^ = 32.89, *p* = 0.15, *I*^2^ = 76%; 3–6 months: *χ*^2^ = 6.96, *p* = 0.14, *I*^2^ = 43%) effectively reduced within-group heterogeneity, suggesting these two factors are partial sources of heterogeneity in MoCA outcomes.

**Table 4:** Subgroup analysis profiles of MoCA scores.

Items	No. of included studies	Heterogeneity (*I*^2^)	Overall effect (*p*)	Pooled effect sizes			Subgroup differences (*p* for interaction)
				MD	Lower 95% CI	Upper 95% CI	
Total							
MoCA	16	80	<0.00001	2.21	1.63	2.80	
Subgroup analysis by stroke type							
Ischemic stroke	11	68	<0.00001	2.09	1.40	2.79	0.87
Ischemic and hemorrhagic stroke	4	89	<0.00001	2.46	1.26	3.66	
Not reported	1	—	0.0009	2.26	0.93	3.59	
Subgroup analysis by acupuncture technique							
Manual acupuncture	13	78	<0.00001	2.27	1.65	2.89	0.76
Electroacupuncture	3	85	0.03	1.98	0.23	3.74	
Subgroup analysis by acupoint combination							
Intervention using SBP acupoints exclusively	5	71	<0.00001	2.03	1.06	3.00	0.05
Combined with intelligence-enhancing and mind-regulating acupoints	5	71	0.003	1.65	0.58	2.73	
Combined with governor vessel-regulating acupoints	2	68	0.0002	2.81	1.36	4.27	
Combined with scalp acupuncture therapy	4	22	<0.00001	3.06	2.58	3.53	
Subgroup analysis by treatment duration							
Treatment courses shorter than 2 weeks	2	52	<0.00001	2.96	1.73	4.18	0.01
Treatment courses within 1–2 months	9	76	<0.00001	2.56	1.87	3.25	
Treatment courses within 3–6 months	5	43	0.0007	1.25	0.52	1.98	
Subgroup analysis by combination with other therapies							
As a standalone cognitive intervention	2	79	0.16	1.16	−0.46	2.77	0.63
Combined with cognitive-enhancing medications	6	68	<0.00001	2.14	1.26	3.01	
Combined with cognitive training	4	88	<0.00001	2.56	1.38	3.74	
Combined with physical therapy	1	—	0.04	2.13	0.09	4.17	
Combined with at least two additional therapeutic modalities	3	20	<0.00001	2.57	1.63	3.52	

Subgroup analyses of effect sizes demonstrated that the therapeutic effect was significantly influenced by and treatment duration (*χ*^2^ = 8.81, *p* for interaction = 0.01). In contrast, neither stroke type (*χ*^2^ = 0.28, *p* for interaction = 0.87), acupuncture technique (*χ*^2^ = 0.09, *p* for interaction = 0.76), acupoint combination (*χ*^2^ = 7.65, *p* for interaction = 0.05), nor combination with other therapies (*χ*^2^ = 2.56, *p* for interaction = 0.63) showed a significant moderating effect. Analysis of treatment duration revealed that shorter courses were associated with better MoCA outcomes: both courses shorter than 2 weeks (MD = 2.96, 95% CI: 1.73–4.18) and 1–2 months (MD = 2.56, 95% CI: 1.87–3.25) significantly outperformed the 3–6-month duration (MD = 1.25, 95% CI: 0.52–1.98). This pattern is consistent with the MMSE outcomes observed in our analysis.

Regarding treatment combinations, SBP acupuncture administered as a standalone cognitive intervention showed no statistically significant effect (MD = 1.16, 95% CI: −0.46 to −2.77, *Z* = 1.40, *p* = 0.16), though this finding requires cautious interpretation due to the small sample size (*n* = 2). In contrast, significantly better MoCA improvements were observed when SBP acupuncture was combined with cognitive-enhancing medications (MD = 2.14, 95% CI: 1.26–3.01), cognitive training (MD = 2.56, 95% CI: 1.38–3.74), physical therapy (MD = 2.13, 95% CI: 0.09–4.17), or at least two additional therapeutic modalities (MD = 2.57, 95% CI: 1.63–3.52). These findings align with the corresponding results from the MMSE analysis.

#### 3.3.4 ADL scores

**Figure 6:**
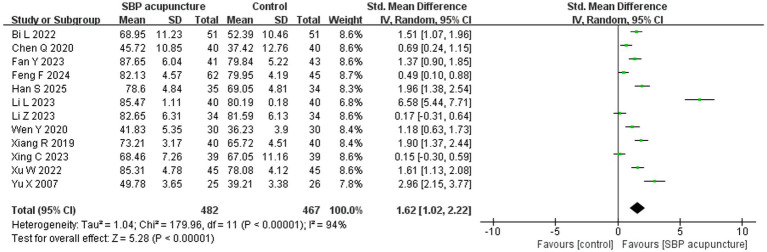
Forest plot of ADL scores.

Twelve studies (19–21, 23, 24, 28, 31, 32, 34, 35, 38, 39) involving 949 patients assessed ADL using either the Barthel index (BI), Modified Barthel index (MBI), or other ADL scales. Significant heterogeneity was observed among the studies (*χ*^2^ = 179.96, *p* < 0.00001, *I*^2^ = 94%), and a random-effects model was therefore applied. The meta-analysis demonstrated that SBP acupuncture significantly improved ADL scores compared with control interventions (SMD = 1.62, 95% CI: 1.02–2.22, *Z* = 5.28, *p* < 0.00001; Figure 6).

Subgroup analyses were conducted across five predefined dimensions: scale type, stroke type, acupuncture technique, acupoint combination, and combination with other therapies (Table 5). The results revealed that none of these prespecified subgroups substantially reduced the observed heterogeneity, suggesting the presence of other unexplained sources of variation.

**Table 5:** Subgroup analysis profiles of ADL scores.

Items	No. of included studies	Heterogeneity (*I*^2^)	Overall effect (*p*)	Pooled effect sizes			Subgroup differences (*p* for interaction)
				MD	Lower 95% CI	Upper 95% CI	
Total							
ADL	12	94	<0.00001	1.62	1.02	2.22	
Subgroup analysis by stroke type							
Ischemic stroke	6	89	<0.00001	1.45	0.84	2.05	0.82
Ischemic and hemorrhagic stroke	4	98	0.02	2.03	0.27	3.79	
Not reported	2	71	<0.00001	1.55	0.84	2.25	
Subgroup analysis by acupuncture technique							
Manual acupuncture	10	94	<0.00001	1.74	1.07	2.41	0.47
Electroacupuncture	2	96	0.25	1.04	−0.73	2.81	
Subgroup analysis by acupoint combination							
Intervention using SBP acupoints exclusively	5	91	0.01	0.86	0.17	1.55	0.16
Combined with intelligence-enhancing and mind-regulating acupoints	1	—	<0.00001	1.51	1.07	1.96	
Combined with governor vessel-regulating acupoints	2	92	0.02	2.04	0.3	3.79	
Combined with scalp acupuncture therapy	4	97	0.002	2.54	0.97	4.11	
Subgroup analysis by combination with other therapies							
As a standalone cognitive intervention	1	—	0.49	0.17	−0.31	0.64	<0.00001
Combined with cognitive-enhancing medications	4	73	<0.00001	1.98	1.42	2.55	
Combined with cognitive training	5	97	0.004	1.89	0.61	3.16	
Combined with at least two additional therapeutic modalities	2	85	0.007	1.11	0.3	1.91	
Subgroup analysis by scale types							
Assessed by MBI scales	5	97	0.004	2.23	0.71	3.75	0.21
Assessed by BI scales	5	89	0.0008	1.1	0.46	1.74	
Assessed by ADL scales	2	29	<0.00001	1.69	1.27	2.12	

Further analyses revealed that the therapeutic effect was significantly influenced by combination with other therapies (*χ*^2^ = 13.61, *p* for interaction <0.00001), while no significant moderating effects were found for acupuncture technique (*χ*^2^ = 0.52, *p* for interaction = 0.47), acupoint combination (*χ*^2^ = 5.15, *p* for interaction = 0.16), stroke type (*χ*^2^ = 0.39, *p* for interaction = 0.82), or assessment scale type (*χ*^2^ = 25.32, *p* for interaction = 0.21). SBP acupuncture as a standalone cognitive intervention, based on a single study, showed no statistically significant effect on ADL (SMD = 0.17, 95% CI: −0.31 to −0.64, *Z* = 0.69, *p* = 0.49). However, when integrated into combination therapies, SBP acupuncture produced substantially better ADL outcomes: combined with cognitive-enhancing medications (SMD = 1.98, 95% CI: 1.42–2.55), with cognitive training (SMD = 1.89, 95% CI: 0.61–3.16), and with multi-modal approaches (SMD = 1.11, 95% CI: 0.30–1.91), a pattern consistent with the MMSE and MoCA outcomes.

#### 3.3.5 Sensitivity analyses

**Figure 7:**
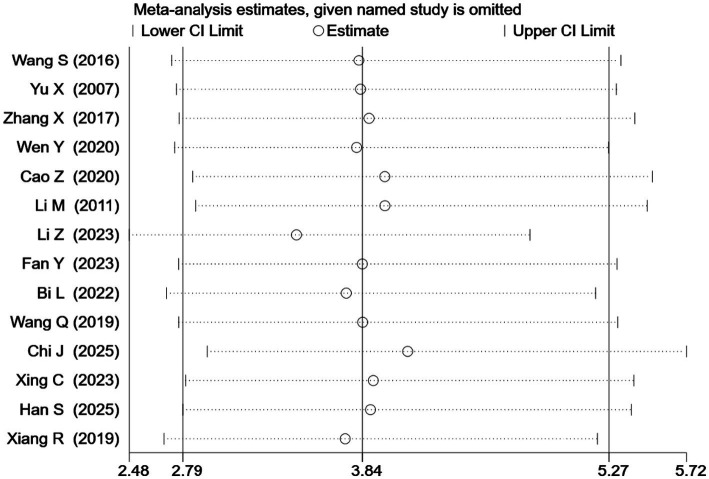
Forest plot of clinical effective rate in leave-one-out sensitivity analysis.

**Figure 8:**
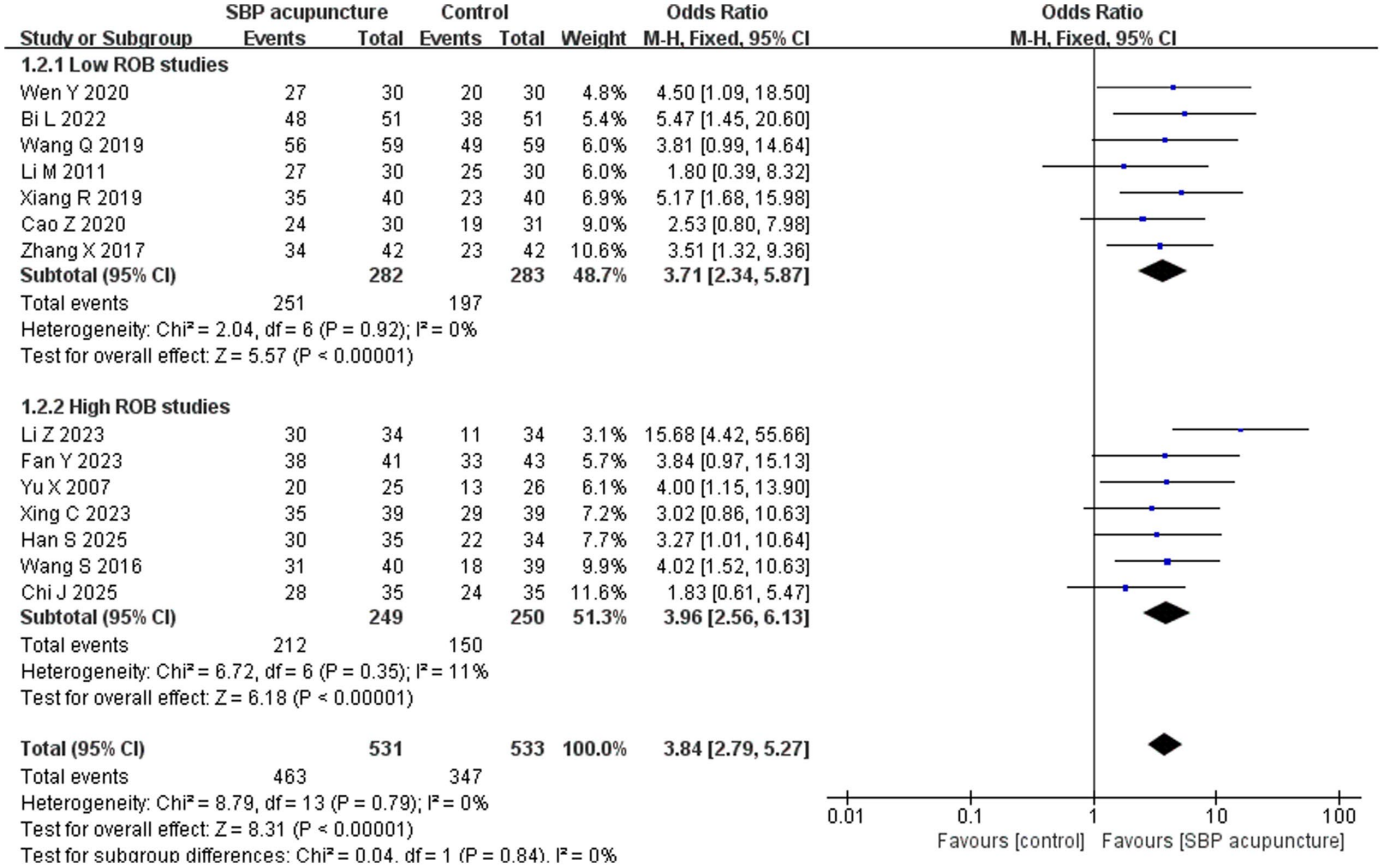
Forest plot of clinical effective rate in stratified analysis by risk of bias.

For the clinical effective rate, the results remained consistent when the OR was replaced with risk ratio (RR = 1.34, 95% CI: 1.25–1.44, *Z* = 8.28, *p* < 0.00001) or risk difference (RD = 0.22, 95% CI: 0.17–0.27, *Z* = 9.09, *p* < 0.00001) for pooling (Table 6). Additionally, the results remained qualitatively unchanged after sequentially omitting individual studies (Figure 7). The robustness of the results was further supported by excluding studies (34, 38) that insufficiently reported the diagnostic criteria for PSCI (OR = 3.80, 95% CI: 2.72–5.31, *Z* = 7.82, *p* < 0.00001; Table 6). Finally, stratified analysis by risk of bias revealed consistent pooled results between low-risk studies (OR = 3.71, 95% CI: 2.34–5.87, *Z* = 5.57, *p* < 0.00001) and high-risk studies (OR = 3.96, 95% CI: 2.56–6.13, *Z* = 6.18, *p* < 0.00001), with no significant interaction (*χ*^2^ = 0.04, *p* for interaction = 0.84; Figure 8). Collectively, these sensitivity analyses confirm the robustness of the clinical effective rate for SBP acupuncture in PSCI.

**Table 6:** Sensitivity analysis of clinical effective rate.

Method of sensitivity analysis		Mean	Lower 95% CI	Upper 95% CI	Heterogeneity test			Meta-analysis	
					*χ* ^2^	*p*	*I* ^2^	*Z*	*p*
Changing effect measures	RR	1.34	1.25	1.44	24.68	0.03	47%	8.28	<0.00001
	RD	0.22	0.17	0.27	23.08	0.04	44%	9.09	<0.00001
Excluding studies that insufficiently reported the diagnostic criteria for PSCI (34, 38)		3.80	2.72	5.31	8.73	0.65	0%	7.82	<0.00001

**Figure 9:**
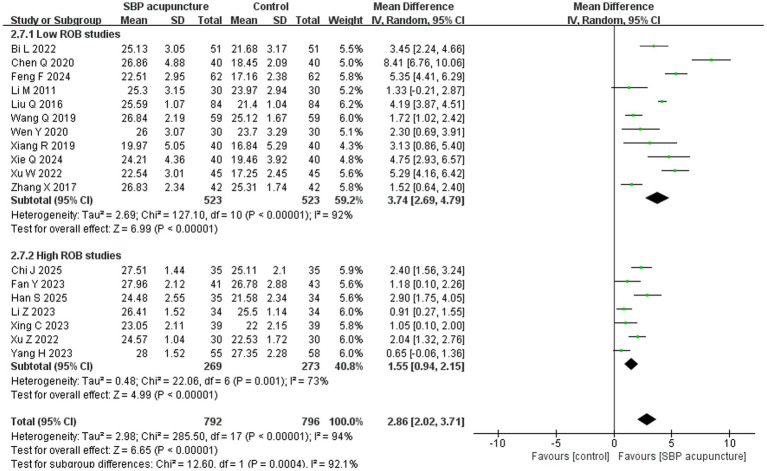
Forest plot of MMSE in stratified analysis by risk of bias.

For MMSE, the results remained consistent when the pooled effect was estimated using SMD (SMD = 1.19, 95% CI: 0.80–1.57, *Z* = 6.02, *p* < 0.00001; Supplementary Table 9) or a fixed-effects model (MD = 2.90, 95% CI: 2.71–3.09, *Z* = 30.02, *p* < 0.00001; Supplementary Table 9), as well as when individual studies were sequentially omitted (Supplementary Figure 1) or studies with insufficient diagnostic reporting for PSCI (23, 38) were excluded (MD = 2.84, 95% CI: 1.92–3.76, *Z* = 6.05, *p* < 0.00001; Supplementary Table 9). Furthermore, stratified analysis by risk of bias revealed consistent pooled results between low-risk studies (MD = 3.74, 95% CI: 2.69–4.79, *Z* = 6.99, *p* < 0.00001) and high-risk studies (MD = 1.55, 95% CI: 0.94–2.15, *Z* = 4.99, *p* < 0.00001); however, high ROB studies appeared to underestimate the pooled effect on MMSE of SBP acupuncture for PSCI (*χ*^2^ = 12.6, *p* for interaction = 0.0004; Figure 9). Collectively, these sensitivity analyses confirm the robustness of the MMSE results for SBP acupuncture in PSCI.

**Figure 10:**
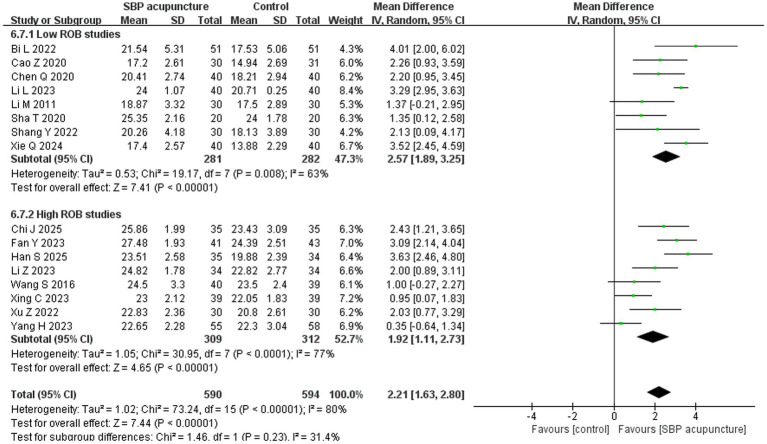
Forest plot of MoCA in stratified analysis by risk of bias.

For MoCA, the results remained consistent when the pooled effect was estimated using SMD (SMD = 0.96, 95% CI: 0.62–1.29, *Z* = 5.65, *p* < 0.00001; Supplementary Table 9) or a fixed-effects model (MD = 2.61, 95% CI: 2.38–2.83, *Z* = 22.35, *p* < 0.00001; Supplementary Table 9), as well as when individual studies were sequentially omitted (Supplementary Figure 1) or studies with insufficient diagnostic reporting for PSCI (38) were excluded (MD = 2.15, 95% CI: 1.52–2.77, *Z* = 6.73, *p* < 0.00001; Supplementary Table 9). Furthermore, stratified analysis by risk of bias revealed consistent pooled results between low-risk studies (MD = 2.57, 95% CI: 1.89–3.25, *Z* = 7.41, *p* < 0.00001) and high-risk studies (MD = 1.92, 95% CI: 1.11–2.73, *Z* = 4.65, *p* < 0.00001), with no significant interaction (*χ*^2^ = 1.46, *p* for interaction = 0.23; Figure 10). Collectively, these sensitivity analyses confirm the robustness of the MoCA results for SBP acupuncture in PSCI.

**Figure 11:**
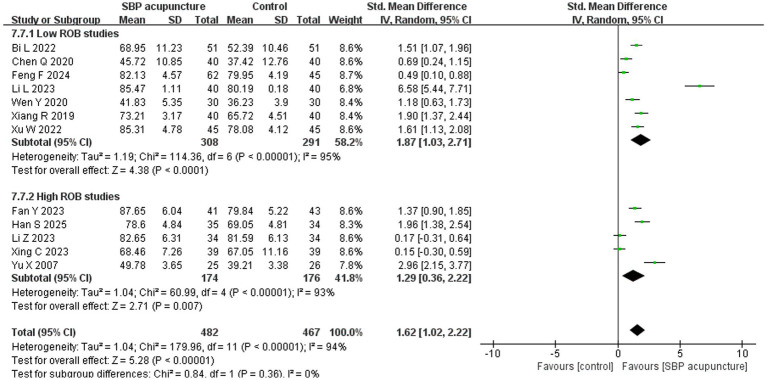
Forest plot of ADL in stratified analysis by risk of bias.

For ADL, the results remained consistent when the pooled effect was estimated using MD (MD = 6.71, 95% CI: 4.88–8.55, *Z* = 7.16, *p* < 0.00001; Supplementary Table 9) or a fixed-effects model (SMD = 1.17, 95% CI: 1.03–1.32, *Z* = 15.72, *p* < 0.00001; Supplementary Table 9), as well as when individual studies were sequentially omitted (Supplementary Figure 1) or studies with insufficient diagnostic reporting for PSCI (23, 34, 38) were excluded (SMD = 1.86, 95% CI: 1.04–2.67, *Z* = 4.48, *p* < 0.00001; Supplementary Table 9). Furthermore, stratified analysis by risk of bias revealed consistent pooled results between low-risk studies (SMD = 1.87, 95% CI: 1.03–2.71, *Z* = 4.38, *p* < 0.00001) and high-risk studies (SMD = 1.29, 95% CI: 0.36–2.22, *Z* = 2.71, *p* = 0.007), with no significant interaction (*χ*^2^ = 0.84, *p* for interaction = 0.36; Figure 11). Collectively, these sensitivity analyses confirm the robustness of the ADL results for SBP acupuncture in PSCI.

#### 3.3.6 Profile of adverse events

Nine studies (19, 20, 24, 25, 27, 30, 33, 41, 42) reported adverse events, while one study (37) mentioned their occurrence but provided no specific data. In the study by Wang et al. (30), 17 adverse reactions were documented (eight in the acupuncture group), including nausea, vomiting, and dizziness; however, no causal analysis was provided. Li (25) reported two cases of dizziness and nausea, which were considered potentially drug-related. Three studies (19, 20, 24) documented four minor acupuncture-related adverse events (subcutaneous hemorrhage, fainting during acupuncture, and needle sticking), all of which were transient and did not interrupt treatment. The remaining four studies (27, 33, 41, 42) reported no observable adverse events. Collectively, these findings suggest that SBP acupuncture has a favorable safety profile.

#### 3.3.7 Publication bias analyses

Assessment of publication bias revealed symmetrical funnel plots (Supplementary Figure 2) with non-significant Egger’s test results for clinical effective rate (*p* = 0.776), MMSE (*p* = 0.555), and MoCA (*p* = 0.052), indicating low publication bias risk.

**Figure 12:**
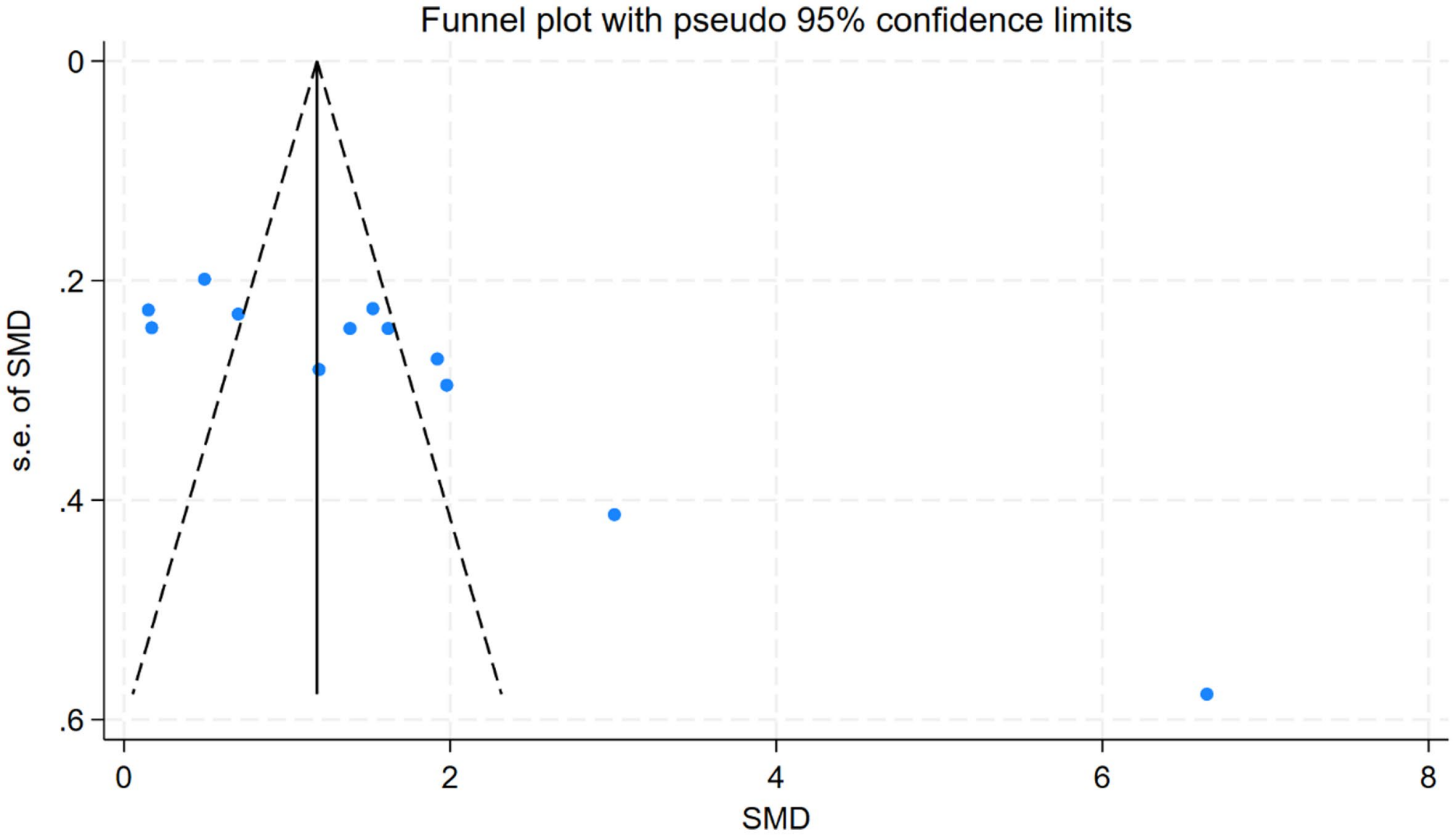
Funnel plot of ADL outcomes.

In contrast, ADL outcomes showed significant funnel plot asymmetry (Egger’s test *p* < 0.001), suggesting the existence of potential publication bias (Figure 12); however, this finding should be interpreted with caution given the substantial heterogeneity across studies. We performed precision-effect test and precision-effect estimate with standard errors (PET-PEESE) analyses to adjust for small-study effects. The PET analysis yielded a statistically significant corrected effect (SMD = −2.75, 95% CI: −3.87 to −1.63, *Z* = −4.81, *p* < 0.0001), while the PEESE analysis, which provides a more reliable estimate in the presence of small-study effects, was no longer statistically significant (SMD = −0.20, 95% CI: −0.72 to 0.33, *Z* = −0.73, *p* = 0.467). Collectively, these findings suggest that the initial pooled effect may have been overestimated due to small-study effects and publication bias.

## 4 Discussion

This meta-analysis of 24 RCTs demonstrated that SBP acupuncture may be effective for treating PSCI and well tolerated, with potential benefits on both MMSE and MoCA scores, although high heterogeneity was observed specifically for these outcomes across the included studies. These observed therapeutic effects of SBP acupuncture in PSCI may be attributed to multiple mechanisms, ranging from immediate antihypertensive effects (46) to sustained regulation of inflammatory reactions, neurotrophic factors, brain functional networks, and cerebral blood flow (47–49).

The robustness of these findings was further supported by both subgroup analyses based on stroke type and four predefined intervention components, and sensitivity analyses, including alternating effect measures and statistical models, sequentially omitting individual studies, excluding studies with insufficient diagnostic reporting for PSCI, and performing stratified analyses by risk of bias. After identifying combination therapies as a partial source of heterogeneity in MMSE scores, and acupoint selection and treatment duration as partial sources of heterogeneity in MoCA scores, the pooled effects of SBP acupuncture on MMSE and MoCA scores in PSCI remained statistically significant within each subgroup. Sensitivity analyses consistently corroborated these findings, yielding unchanged results.

Moreover, subgroup analyses based on therapeutic combinations demonstrated consistently superior efficacy across these measured outcomes when SBP acupuncture was integrated into a combination therapy regimen. This finding aligns with previous reports (9, 14, 50, 51), supporting the inclusion of acupuncture as an adjunctive therapy within a comprehensive treatment regimen. This suggests a synergistic interaction between acupuncture and other cognitive therapies for improving cognitive function. Notably, this study did not observe substantial benefit from treatment regimens extending beyond 3 months, highlighting that longer treatment durations may not yield additional gains. Given the limited evidence and considerable heterogeneity in treatment durations of current acupuncture therapies (12), this study underscores the need for further research to determine the optimal course for cognitive improvement in PSCI.

This review also evaluated the effect of SBP acupuncture on ADL in patients with PSCI. Despite unresolved high heterogeneity, the meta-analysis revealed that SBP acupuncture may yield benefits on ADL outcomes in PSCI. Subgroup and sensitivity analyses yielded unchanged results; however, the certainty of this finding is undermined by the presence of publication bias. PET-PEESE adjustment for publication bias revealed that this finding may be driven by small-study effects. The PEESE estimate, which is more reliable in the presence of publication bias, was no longer statistically significant. Therefore, the potential benefits of SBP acupuncture on ADL in PSCI remain uncertain, and the available evidence does not support a definitive conclusion.

The certainty of evidence was assessed using the Grading of Recommendations, Assessment, Development, and Evaluation (GRADE) framework (Table 7). The evidence for the clinical effective rate was rated as moderate, downgraded one level due to a serious risk of bias, while evidence for MMSE and MoCA outcomes was rated as low, downgraded for both serious risk of bias and substantial heterogeneity. The evidence for the secondary ADL outcome was rated as very low, downgraded further for imprecision and publication bias. Consequently, the certainty of evidence is largely affected by serious risk of bias across outcomes.

**Table 7:** GRADE evidence profile: SBP acupuncture for PSCI.

No. of studies	Design	ROB	Inconsistency	Indirectness	Imprecision	Other considerations	No. of patients		Effect		Certainty	Importance
							SBP acupuncture	Control	Relative (95% CI)	Absolute (95% CI)		
Clinical effective effect												
14	RCT	Serious^a^	Not serious	Not serious	Not serious	None	531	533	OR 3.84 (2.79 to 5.27)	226 more per 1,000 (188 to 257 more per 1,000)	⨁⨁⨁◯ Moderate	Critical
MMSE												
18	RCT	Serious^b^	Serious^c^	Not serious	Not serious	None	792	796	—	MD 2.86 (2.02 to 3.71)	⨁⨁◯◯ Low	Critical
MoCA												
16	RCT	Serious^a^	Serious^c^	Not serious	Not serious	None	590	594	—	MD 2.21 (1.63 to 2.80)	⨁⨁◯◯ Low	Critical
ADL												
12	RCT	Serious^b^	Serious^c^	Not serious	Serious^d^	Publication bias^e^	482	467	—	SMD 1.62 (1.02 to 2.22)	⨁◯◯◯ Very low	Important

a Insufficient allocation concealment, blinding of participants and personnel are not adopted or described, incomplete outcome data, unclear selecting reporting, other bias.

b Insufficient allocation concealment, blinding of participants and personnel are not adopted or described, incomplete outcome data, unclear selecting reporting.

c Considerable statistical heterogeneity.

d The result was no longer statistically significant after PET-PEESE adjustment.

e Asymmetrical funnel plot with Egger’s test *p* < 0.001.

Although our stratified analyses by risk of bias revealed that high ROB studies appeared to underestimate the pooled effect on MMSE, these findings should still be interpreted with caution given the poor methodological quality across the included studies. These concerns are reflected in the prevalent methodological limitations listed below: (1) inadequate allocation concealment due to insufficient reporting; (2) limited blinding implementation, with no acupuncturist blinding and only a few participant/assessor blinding; (3) incomplete outcome data handling, with two studies using complete-case analysis; (4) a high risk of selective reporting, with six studies employing per-protocol analysis; (5) a lack of prospective registration in most studies, hindering comprehensive outcome assessment; (6) a lack of follow-up assessments in most studies, making long-term efficacy uncertain; and (7) the exclusive conduct of studies in China with predominantly Chinese-language publications, introducing potential geographical and language bias.

Thus, while current evidence suggests the potential clinical value of SBP acupuncture for PSCI, conclusions remain preliminary. To strengthen the evidence base for SBP acupuncture in managing PSCI, future research should consider the following priorities: (1) Conduct large-scale, multicenter RCTs across diverse geographic regions to improve the generalizability of findings; (2) enhance methodological rigor through standardized implementation of randomization, allocation concealment, and blinding procedures; (3) implement prospective trial registration as a standard practice to improve transparency and prevent selective outcome reporting; (4) establish systematic follow-up assessments to evaluate the long-term sustainability of therapeutic benefits; and (5) utilize advanced statistical modeling techniques to better delineate sources of treatment effects and identify potential effect modifiers.

## 5 Conclusion

Our study indicates that SBP acupuncture may be effective in treating PSCI, with moderate certainty for the clinical effective rate. Improvements in MMSE and MoCA scores suggest that SBP acupuncture may offer cognitive benefits in PSCI. More favorable outcomes were observed when SBP acupuncture was administered as part of a combination therapy regimen or in treatment courses shorter than 3 months. However, the certainty of these cognitive findings is low due to high heterogeneity and pervasive methodological bias. Benefits for ADL outcomes appear uncertain, particularly given the presence of publication bias. Therefore, while these findings are promising, they remain preliminary and should be interpreted with caution before application to clinical practice. Further large-scale, multicenter, registered RCTs with rigorous methodologies and long-term follow-up are needed to validate these preliminary results and establish more definitive evidence regarding the efficacy and optimal application of SBP acupuncture for PSCI.

## Data Availability

The original contributions presented in the study are included in the article/Supplementary material, further inquiries can be directed to the corresponding author.

## Glossary

AD: Alzheimer’s disease
ADL: Activities of daily living
BI: Barthel index
CBM: China biology medicine disc
CC analysis: Complete-case analysis
CI: Confidence interval
CNKI: Chinese National Knowledge Infrastructure
GRADE: Grading of Recommendations Assessment, Development and Evaluation
MBI: Modified Barthel index
MD: Mean difference
MeSH: Medical subject heading
MMSE: Mini-Mental State Examination
MoCA: Montreal Cognitive Assessment
OR: Odds ratio
PET-PEESE: Precision-Effect Test and Precision-Effect Estimate with Standard Errors
PICOS: Population, Intervention, Comparison, Outcomes and Study
PP analysis: Per-protocol analysis
PRISMA: Preferred Reporting Items for Systematic Reviews and Meta-analyses
PSCI: Post-stroke cognitive impairment
RCTs: Randomized controlled trials
RD: Risk difference
ROB: Risk of bias
RR: Risk ratio
SBP: Skull base-peripheral
SMD: Standardized mean difference
TCM: Traditional Chinese medicine
