# Supplementary material for: Effectiveness and safety of skull base-peripheral acupuncture for post-stroke cognitive impairment: a systematic review and meta-analysis of randomized controlled trials
Source: Front Neurol. 2026 Apr 30;17:1790835. doi: 10.3389/fneur.2026.1790835 (PMC13171405; doi:10.3389/fneur.2026.1790835)
Supplement: Supplementary file 1 [file Data_Sheet_1.DOCX]

**Supplementary Appendix**

**Table 1: Search Strategy for PubMed**

| No. | Search Items |
| --- | --- |
| #1 | "Cognitive dysfunction"[Mesh] OR ("cognition disorder*" OR "cognitive complaints" OR "cognitive decline*" OR "cognitive defect*" OR "cognitive deficiency" OR "cognitive deficit" OR "cognitive difficulties" OR "cognitive disability" OR "cognitive disorder*" OR "cognitive disturbance" OR "cognitive dysfunctions" OR "cognitive impairment*" OR "cognitive problems" OR "overinclusion" OR "response interference" OR "Mild Cognitive Impairment*" OR "Mental Deterioration*" ) |
| #2 | "Stroke"[Mesh] OR ("Strokes" OR "Cerebrovascular Accident*" OR "Cerebral Stroke*" OR "Cerebrovascular Apoplexy" OR "Brain Vascular Accident*" OR "Cerebrovascular Stroke*" OR "Apoplexy" OR "CVA" OR "CVAs" OR "Acute Stroke*" OR "Acute Cerebrovascular Accident*" OR "acute cerebrovascular lesion" OR "acute focal cerebral vasculopathy" OR "apoplectic stroke" OR "apoplexia" OR "brain attack" OR "brain insult" OR "cerebral insult" OR "cerebral vascular accident" OR "cerebral vascular insufficiency" OR "cerebrovascular failure" OR "cerebrovascular injury" OR "cerebrovascular insufficiency" OR "cerebrovascular insult" OR "cryptogenic stroke" OR "insultus cerebralis" OR "thrombotic stroke" ) |
| #3 | "acupuncture"[Mesh] OR "acupuncture therapy"[Mesh] OR "Acupuncture Points"[Mesh] OR "Pharmacopuncture" OR "Acupuncture Treatment*" OR "Pharmacoacupuncture Treatment" OR "Pharmacoacupuncture Therapy" OR "Acupotomy" OR "Acupotomies" OR "Acupuncture Point" OR "Acupoint*" OR "Electroacupuncture" OR "Scalp Acupuncture" OR "meridian point" OR "acupuncture-moxibustion therapy" |
| #4 | #1 AND #2 AND #3 |

**Table 2: Search Strategy for The Cochrane Library**

| No. | Search Items |
| --- | --- |
| #1 | MeSH descriptor: [Cognitive Dysfunction] explode all trees |
| #2 | (Cognitive Dysfunctions):ti,ab,kw OR (Cognitive Disorder*):ti,ab,kw OR (Cognitive Impairment*):ti,ab,kw OR (Mild Cognitive Impairment*):ti,ab,kw OR (Cognitive Decline*):ti,ab,kw OR (Mental Deterioration*):ti,ab,kw OR (cognitive deficit):ti,ab,kw OR (cognition disorder*):ti,ab,kw OR (cognitive complaints):ti,ab,kw OR (cognitive defect*):ti,ab,kw OR (cognitive deficiency):ti,ab,kw OR (cognitive difficulties):ti,ab,kw OR (cognitive disability):ti,ab,kw OR (cognitive disturbance):ti,ab,kw OR (cognitive problems):ti,ab,kw OR (overinclusion):ti,ab,kw OR (response interference):ti,ab,kw OR (slight cognition disfunction):ti,ab,kw |
| #3 | MeSH descriptor: [Stroke] explode all trees |
| #4 | (Strokes):ti,ab,kw OR (CVA):ti,ab,kw OR (Apoplexy):ti,ab,kw OR (Cerebrovascular Accident*):ti,ab,kw OR (Cerebrovascular Stroke*):ti,ab,kw OR (Brain Vascular Accident*):ti,ab,kw OR (Cerebral Stroke*):ti,ab,kw OR (Cerebrovascular Apoplexy):ti,ab,kw OR (CVAs):ti,ab,kw OR (Acute Stroke*):ti,ab,kw OR (Acute Cerebrovascular Accident*):ti,ab,kw OR (acute cerebrovascular lesion):ti,ab,kw OR (acute focal cerebral vasculopathy):ti,ab,kw OR (apoplectic stroke):ti,ab,kw OR (apoplexia):ti,ab,kw OR (brain accident):ti,ab,kw OR (brain attack):ti,ab,kw OR (brain blood flow disturbance):ti,ab,kw OR (brain insult*):ti,ab,kw OR (cerebral apoplexia):ti,ab,kw OR (cerebral insult):ti,ab,kw OR (cerebral vascular accident):ti,ab,kw OR (cerebral vascular insufficiency):ti,ab,kw OR (cerebrovascular failure):ti,ab,kw OR (cerebrovascular insufficiency):ti,ab,kw OR (cerebrovascular insult):ti,ab,kw OR (cerebrum vascular accident):ti,ab,kw OR (cryptogenic stroke):ti,ab,kw OR (insultus cerebralis):ti,ab,kw OR (ischaemic seizure):ti,ab,kw OR (ischemic seizure):ti,ab,kw OR (thrombotic stroke):ti,ab,kw OR (cerebrovascular injury):ti,ab,kw OR (cerebrovascular arrest):ti,ab,kw |
| #5 | MeSH descriptor: [Acupuncture] explode all trees |
| #6 | MeSH descriptor: [Acupuncture Therapy] explode all trees |
| #7 | MeSH descriptor: [Acupuncture Points] explode all trees |
| #8 | (Pharmacopuncture):ti,ab,kw OR (Acupuncture Point):ti,ab,kw OR (Acupuncture Treatment*):ti,ab,kw OR (shonishin):ti,ab,kw OR (Pharmacoacupuncture Treatment):ti,ab,kw OR (Pharmacoacupuncture Therapy):ti,ab,kw OR (Acupotomy):ti,ab,kw OR (Acupotomies):ti,ab,kw OR (Acupoint*):ti,ab,kw OR (Electroacupuncture):ti,ab,kw OR (Scalp Acupuncture):ti,ab,kw OR (meridian point):ti,ab,kw OR (acupuncture-moxibustion therapy):ti,ab,kw |
| #9 | #1 OR #2 |
| #10 | #3 OR #4 |
| #11 | #5 OR #6 OR #7 OR #8 |
| #12 | #9 AND #10 AND #11 in Trials |

**Table 3: Search Strategy for Web of Science**

| No. | Search Items |
| --- | --- |
| #1 | TS=Cognitive dysfunction* OR Cognitive Disorder* OR Cognitive Impairment* OR Mild Cognitive Impairment* OR Cognitive Decline* OR Mental Deterioration* OR cognitive deficit OR cognition disorder* OR cognitive complaints OR cognitive defect* OR cognitive deficiency OR cognitive difficulties OR cognitive disability OR cognitive disturbance OR cognitive problems OR overinclusion OR response interference OR slight cognition disfunction |
| #2 | TS=Stroke* OR CVA OR Apoplexy OR Cerebrovascular Accident* OR Cerebrovascular Stroke* OR Brain Vascular Accident* OR Cerebral Stroke* OR Cerebrovascular Apoplexy OR CVAs OR Acute Stroke* OR Acute Cerebrovascular Accident* OR acute cerebrovascular lesion OR acute focal cerebral vasculopathy OR apoplectic stroke OR apoplexia OR brain accident OR brain attack OR brain blood flow disturbance OR brain insult* OR cerebral apoplexia OR cerebral insult OR cerebral vascular accident OR cerebral vascular insufficiency OR cerebrovascular failure OR cerebrovascular insufficiency OR cerebrovascular insult OR cerebrum vascular accident OR cryptogenic stroke OR insultus cerebralis OR ischaemic seizure OR ischemic seizure OR thrombotic stroke OR cerebrovascular injury OR cerebrovascular arrest |
| #3 | TS=Acupuncture OR Acupuncture Therapy OR Acupuncture Point* OR Pharmacopuncture OR Acupuncture Treatment* OR shonishin OR Pharmacoacupuncture Treatment OR Pharmacoacupuncture Therapy OR Acupotomy OR Acupotomies OR Acupoint* OR Electroacupuncture (主题) OR Scalp Acupuncture OR meridian point OR acupuncture-moxibustion therapy |
| #4 | #1 AND #2 AND #3 |

**Table 4: Search Strategy for Embase**

| No. | Search Items |
| --- | --- |
| #1 | 'cognitive defect'/exp OR ('cognition disorder*' OR 'cognitive complaints' OR 'cognitive decline*' OR 'cognitive defect*' OR 'cognitive deficiency' OR 'cognitive deficit' OR 'cognitive difficulties' OR 'cognitive disability' OR 'cognitive disorder*' OR 'cognitive disturbance' OR 'cognitive dysfunction*' OR 'cognitive impairment*' OR 'cognitive problems' OR 'overinclusion' OR 'response interference' OR 'Mild Cognitive Impairment*' OR 'Mental Deterioration*' OR 'slight cognition disfunction') |
| #2 | 'cerebrovascular accident'/exp OR ('acute cerebrovascular lesion' OR 'acute focal cerebral vasculopathy' OR 'acute stroke*' OR 'apoplectic stroke' OR 'apoplexia' OR 'apoplexy' OR 'brain accident' OR 'brain attack' OR 'brain blood flow disturbance' OR 'brain insult*' OR 'brain vascular accident*' OR 'cerebral apoplexia' OR 'cerebral insult' OR 'cerebral stroke*' OR 'cerebral vascular accident' OR 'cerebral vascular insufficiency' OR 'cerebrovascular arrest' OR 'cerebrovascular failure' OR 'cerebrovascular injury' OR 'cerebrovascular insufficiency' OR 'cerebrovascular insult' OR 'cerebrum vascular accident' OR 'cryptogenic stroke' OR 'CVA' OR 'insultus cerebralis' OR 'ischaemic seizure' OR 'ischemic seizure' OR 'stroke*' OR 'thrombotic stroke' OR 'Cerebrovascular Accidents' OR 'Cerebrovascular Apoplexy' OR 'CVAs' OR 'Acute Cerebrovascular Accident*' OR 'Cerebrovascular Stroke*') |
| #3 | 'acupuncture'/exp OR 'acupuncture point'/exp OR 'acupuncture therapy' OR 'shonishin' OR 'acupoint*' OR 'acupuncture points' OR 'Pharmacopuncture' OR 'Acupuncture Treatment*' OR 'Pharmacoacupuncture Treatment' OR 'Pharmacoacupuncture Therapy' OR 'Acupotomies' OR 'Acupotomy' OR 'Acupoint*' OR 'Electroacupuncture' OR 'Scalp Acupuncture' OR 'meridian point)' OR 'acupuncture-moxibustion therapy' |
| #4 | #1 AND #2 AND #3 |

**Table 5: Search Strategy for CBM**

| No. | Search Items |
| --- | --- |
| #1 | "ren zhi gong neng zhang ai"[bu jia quan : kuo zhan] huo "qing du ren zhi zhang ai"[chang yong zi duan : zhi neng] huo "qing du shen jing ren zhi zhang ai"[chang yong zi duan : zhi neng] huo "ren zhi jian tui"[chang yong zi duan : zhi neng] huo "jing shen shuai tui"[chang yong zi duan : zhi neng] huo "ren zhi sun hai"[chang yong zi duan : zhi neng] huo "ren zhi zhang ai"[chang yong zi duan : zhi neng] huo ""cognition"[chang yong zi duan : zhi neng] he "disorders""[chang yong zi duan : zhi neng] huo ""cognitive"[chang yong zi duan : zhi neng] he "deficit""[chang yong zi duan : zhi neng] huo ""cognitive"[chang yong zi duan : zhi neng] he "disorder*""[chang yong zi duan : zhi neng] huo "shen jing xing wei zhang ai"[chang yong zi duan : zhi neng] huo "qing du ren zhi gong neng zhang ai"[chang yong zi duan : zhi neng] huo ""mild"[chang yong zi duan : zhi neng] he "cognitive"[chang yong zi duan : zhi neng] he "impairment""[chang yong zi duan : zhi neng] huo ""slight"[chang yong zi duan : zhi neng] he "cognition"[chang yong zi duan : zhi neng] he "dysfunction""[chang yong zi duan : zhi neng] huo "qing du ren zhi gong neng sun hai"[chang yong zi duan : zhi neng] huo "qing du ren zhi gong neng sun shang"[chang yong zi duan : zhi neng] huo "qing du ren zhi sun hai"[chang yong zi duan : zhi neng] huo "qing du ren zhi sun shang"[chang yong zi duan : zhi neng] huo "qing wei ren zhi gong neng sun hai"[chang yong zi duan : zhi neng] |
| #2 | "cu zhong"[bu jia quan : kuo zhan] OR "ji xing cu zhong"[chang yong zi duan : zhi neng] OR "nao cu zhong"[chang yong zi duan : zhi neng] OR "nao zhong feng"[chang yong zi duan : zhi neng] OR "nao xue guan yi wai"[chang yong zi duan : zhi neng] OR "CVA*"[chang yong zi duan : zhi neng] OR "nao xue guan zhong feng"[chang yong zi duan : zhi neng] OR "ji xing nao xue guan yi wai"[chang yong zi duan : zhi neng] OR "ji xing nao cu zhong"[chang yong zi duan : zhi neng] OR "zhong feng"[chang yong zi duan : zhi neng] OR "que xue xing nao cu zhong"[chang yong zi duan : zhi neng] |
| #3 | (((("zhen ci liao fa"[bu jia quan : kuo zhan]) OR "zhen ci xue wei"[bu jia quan : kuo zhan]) OR "dian zhen liao fa"[bu jia quan : kuo zhan]) OR "tou zhen liao fa"[bu jia quan : kuo zhan]) OR "hao zhen"[bu jia quan : kuo zhan] OR "zhen fa"[chang yong zi duan : zhi neng] OR "dian zhen"[chang yong zi duan : zhi neng] OR "Electroacupuncture"[chang yong zi duan : zhi neng] OR "tou pi zhen"[chang yong zi duan : zhi neng] OR "tou zhen"[chang yong zi duan : zhi neng] OR ""Scalp"[chang yong zi duan : zhi neng] AND "Acupuncture""[chang yong zi duan : zhi neng] OR "jing xue"[chang yong zi duan : zhi neng] OR "shu xue"[chang yong zi duan : zhi neng] OR "xue wei"[chang yong zi duan : zhi neng] OR "zhen ci ci ji dian"[chang yong zi duan : zhi neng] OR "Acupoint*"[chang yong zi duan : zhi neng] OR "Point*"[chang yong zi duan : zhi neng] OR "zhen jiu liao fa"[chang yong zi duan : zhi neng] OR "acupuncture"[chang yong zi duan : zhi neng] OR ""acupuncture"[chang yong zi duan : zhi neng] AND "therapy""[chang yong zi duan : zhi neng] OR ""acupuncture-moxibustion"[chang yong zi duan : zhi neng] AND "therapy""[chang yong zi duan : zhi neng] OR ""acupuncture"[chang yong zi duan : zhi neng] AND "point""[chang yong zi duan : zhi neng] OR ""meridian"[chang yong zi duan : zhi neng] AND "point""[chang yong zi duan : zhi neng] OR "ren ti xue wei"[chang yong zi duan : zhi neng] OR "xue dao"[chang yong zi duan : zhi neng] |
| #4 | #1 AND #2 AND #3 |

**Table 6: Search Strategy for CNKI**

| No. | Search Items |
| --- | --- |
| #1 | SU=("ren zhi gong neng zhang ai" + "qing du ren zhi zhang ai" + "qing du shen jing ren zhi zhang ai" + "ren zhi jian tui" + "jing shen shuai tui" + "ren zhi sun hai" + "ren zhi zhang ai"+ "cognition disorders" + "cognitive deficit" + "cognitive disorder*" + "shen jing xing wei zhang ai" + "qing du ren zhi gong neng zhang ai" + "mild cognitive impairment" + "slight cognition dysfunction" + "qing du ren zhi gong neng sun hai" + "qing du ren zhi gong neng sun shang" + "qing du ren zhi sun hai" + "qing du ren zhi sun shang" + "qing wei ren zhi gong neng sun hai") |
| #2 | SU=("cu zhong" + "ji xing cu zhong" + "nao cu zhong" + "nao zhong feng" + "nao xue guan yi wai" + "CVA*" + "nao xue guan zhong feng" + "ji xing nao xue guan yi wai" + "ji xing nao cu zhong" + "zhong feng" + "que xue xing nao cu zhong") |
| #3 | SU=("zhen ci liao fa" + "dian zhen liao fa" + "tou zhen liao fa" + "zhen ci xue wei" + "hao zhen" + "zhen fa" + "dian zhen" + "Electroacupuncture" + "tou pi zhen" + "tou zhen" + "Scalp Acupuncture" + "jing xue" + "shu xue" + "xue wei" + "zhen jiu ci ji dian" + "Acupoint*" + "Point*" + "zhen jiu liao fa" + "acupuncture" + "acupuncture therapy" + "acupuncture-moxibustion therapy" + "acupuncture point" + "meridian point" + "ren ti xue wei" + "xue dao") |
| #4 | #1 AND #2 AND #3 |

**Table 7: Search Strategy for VIP**

| No. | Search Items |
| --- | --- |
| #1 | U=("ren zhi gong neng zhang ai" OR "qing du ren zhi zhang ai" OR "qing du shen jing ren zhi zhang ai" OR "ren zhi jian tui" OR "jing shen shuai tui" OR "ren zhi sun hai" OR "ren zhi zhang ai" OR "cognition disorders" OR "cognitive deficit" OR "cognitive disorder*" OR "shen jing xing wei zhang ai" OR "qing du ren zhi gong neng zhang ai" OR "mild cognitive impairment" OR "slight cognition dysfunction" OR "qing du ren zhi gong neng sun hai" OR "qing du ren zhi gong neng sun shang" OR "qing du ren zhi sun hai" OR "qing du ren zhi sun shang" OR "qing wei ren zhi gong neng sun hai") |
| #2 | U=("cu zhong" OR "ji xing cu zhong" OR "nao cu zhong" OR "nao zhong feng" OR "nao xue guan yi wai" OR "CVA*" OR "nao xue guan zhong feng" OR "ji xing nao xue guan yi wai" OR "ji xing nao cu zhong" OR "zhong feng" OR "que xue xing nao cu zhong") |
| #3 | U=("zhen ci liao fa" OR "dian zhen liao fa" OR "tou zhen liao fa" OR "zhen ci xue wei" OR "hao zhen" OR "zhen fa" OR "dian zhen" OR "Electroacupuncture" OR "tou pi zhen" OR "tou zhen" OR "Scalp Acupuncture" OR "jing xue" OR "shu xue" OR "xue wei" OR "zhen jiu ci ji dian" OR "Acupoint*" OR "Point*" OR "zhen jiu liao fa" OR "acupuncture" OR "acupuncture therapy" OR "acupuncture-moxibustion therapy" OR "acupuncture point" OR "meridian point" OR "ren ti xue wei" OR "xue dao") |
| #4 | #1 AND #2 AND #3 |

**Table 8: Search Strategy for Wanfang**

| No. | Search Items |
| --- | --- |
| #1 | Zhu ti:("ren zhi gong neng zhang ai" OR "qing du ren zhi zhang ai" OR "qing du shen jing ren zhi zhang ai" OR "ren zhi jian tui" OR "jing shen shuai tui" OR "ren zhi sun hai" OR "ren zhi zhang ai" OR "cognition disorders" OR "cognitive deficit" OR "cognitive disorder*" OR "shen jing xing wei zhang ai" OR "qing du ren zhi gong neng zhang ai" OR "mild cognitive impairment" OR "slight cognition dysfunction" OR "qing du ren zhi gong neng sun hai" OR "qing du ren zhi gong neng sun shang" OR "qing du ren zhi sun hai" OR "qing du ren zhi sun shang" OR "qing wei ren zhi gong neng sun hai") |
| #2 | Zhu ti:("cu zhong" OR "ji xing cu zhong" OR "nao cu zhong" OR "nao zhong feng" OR "nao xue guan yi wai" OR "CVA*" OR "nao xue guan zhong feng" OR "ji xing nao xue guan yi wai" OR "ji xing nao cu zhong" OR "zhong feng" OR "que xue xing nao cu zhong") |
| #3 | Zhu ti:("zhen ci liao fa" OR "dian zhen liao fa" OR "tou zhen liao fa" OR "zhen ci xue wei" OR "hao zhen" OR "zhen fa" OR "dian zhen" OR "Electroacupuncture" OR "tou pi zhen" OR "tou zhen" OR "Scalp Acupuncture" OR "jing xue" OR "shu xue" OR "xue wei" OR "zhen jiu ci ji dian" OR "Acupoint*" OR "Point*" OR "zhen jiu liao fa" OR "acupuncture" OR "acupuncture therapy" OR "acupuncture-moxibustion therapy" OR "acupuncture point" OR "meridian point" OR "ren ti xue wei" OR "xue dao") |
| #4 | #1 AND #2 AND #3 |

**Table 9: Sensitivity Analysis of MMSE, MoCA, and ADL**

| Outcomes | Method of Sensitivity Analysis | | Mean | Lower 95% CI | Upper 95% CI | Heterogeneity Test | | | Meta-Analysis | | |
| --- | --- | --- | --- | --- | --- | --- | --- | --- | --- | --- | --- |
|  |  |  |  |  |  | *Χ^2^* | *P* | *I²* | *Z* | | *P* |
| **MMSE** | Changing effect measures | SMD | 1.19 | 0.80 | 1.57 | 209.46 | < 0.00001 | 92% | 6.02 | | < 0.00001 |
|  | Changing statistic models | Fixed-Effects Model | 2.90 | 2.71 | 3.09 | 285.5 | < 0.00001 | 94% | 30.02 | | < 0.00001 |
|  | Excluding studies that insufficiently reported the diagnostic criteria for PSCI  (Feng F 2024, Fan Y 2023) | | 2.84 | 1.92 | 3.76 | 249.12 | < 0.00001 | 94% | 6.05 | | < 0.00001 |
| **MoCA** | Changing effect measures | SMD | 0.96 | 0.62 | 1.29 | 108.98 | < 0.00001 | 86% | 5.65 | | < 0.00001 |
|  | Changing statistic models | Fixed-Effects Model | 2.61 | 2.38 | 2.83 | 73.24 | < 0.00001 | 80% | 22.35 | | < 0.00001 |
|  | Excluding studies that insufficiently reported the diagnostic criteria for PSCI  (Fan Y 2023) | | 2.15 | 1.52 | 2.77 | 72.19 | < 0.00001 | 81% | 6.73 | | < 0.00001 |
| **ADL** | Changing effect measures | MD | 6.78 | 5.11 | 8.44 | 107.14 | < 0.00001 | 90% | 7.99 | < 0.00001 | |
|  | Changing statistic models | Fixed-Effects Model | 1.17 | 1.03 | 1.32 | 179.06 | < 0.00001 | 94% | 15.72 | < 0.00001 | |
|  | Excluding studies that insufficiently reported the diagnostic criteria for PSCI  (Wen Y 2020, Feng F 2024, Fan Y 2023) | | 1.86 | 1.04 | 2.67 | 166.03 | < 0.00001 | 95% | 4.48 | < 0.00001 | |


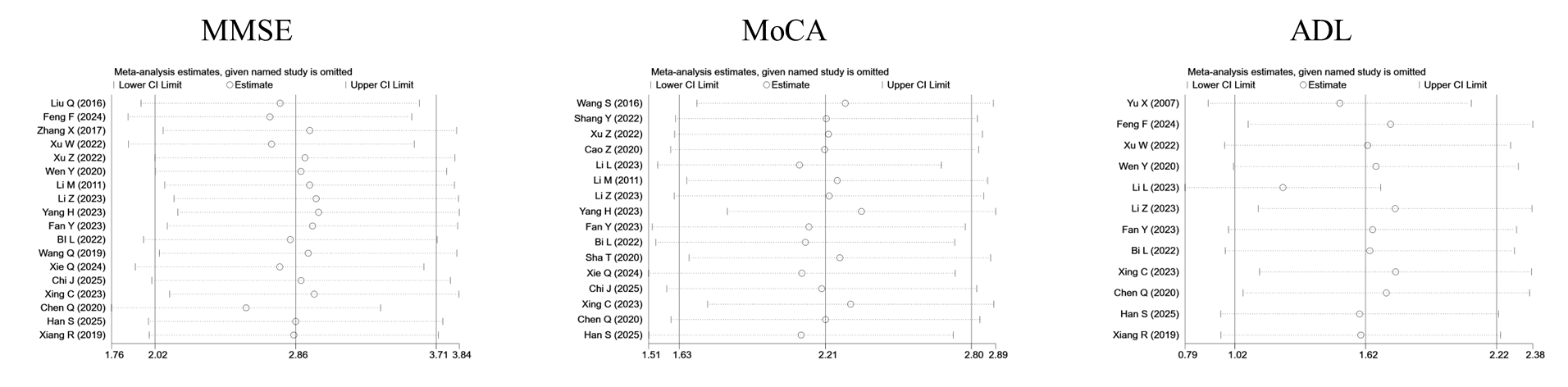


Figure 1: Forest Plot of MMSE, MoCA and ADL Outcomes in Leave-One-Out Sensitivity Analysis


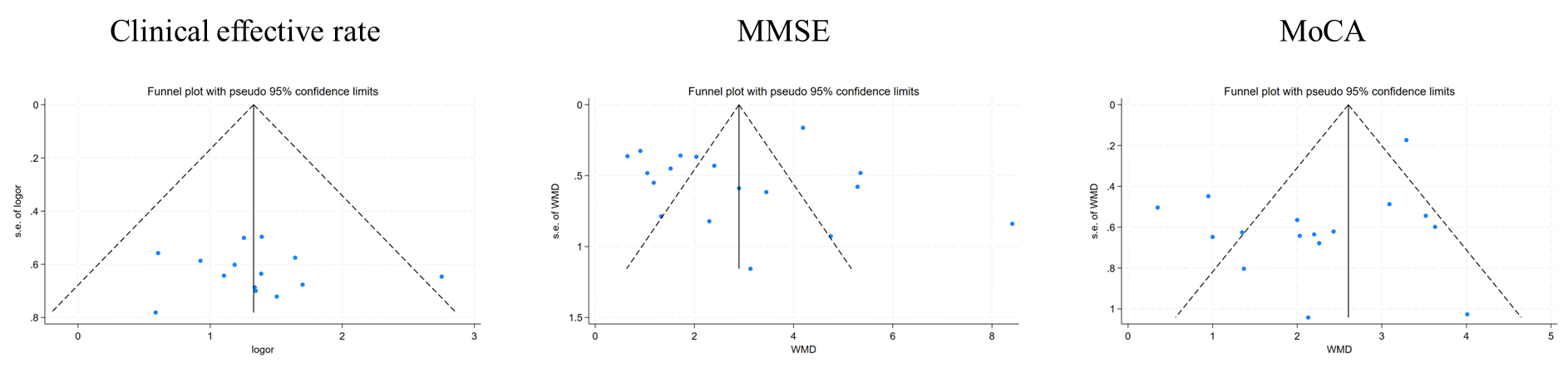


Figure 2: Funnel Plot of Clinical effective rate, MMSE, MoCA Outcomes
